# Pyranose Ring Puckering Thermodynamics for Glycan Monosaccharides Associated with Vertebrate Proteins

**DOI:** 10.3390/ijms23010473

**Published:** 2021-12-31

**Authors:** Olgun Guvench, Devon Martin, Megan Greene

**Affiliations:** 1 Department of Pharmaceutical Sciences and Administration, School of Pharmacy, University of New England, 716 Stevens Avenue, Portland, ME 04103, USA; dmartin11@une.edu (D.M.); mgreene3@une.edu (M.G.); 2 Graduate School of Biomedical Science and Engineering, University of Maine, 5775 Stodder Hall, Orono, ME 04469, USA

**Keywords:** glucose, GlcNAc, galactose, GalNAc, mannose, xylose, fucose, Neu5Ac, glucuronate, iduronate, tetrahydropyran

## Abstract

The conformational properties of carbohydrates can contribute to protein structure directly through covalent conjugation in the cases of glycoproteins and proteoglycans and indirectly in the case of transmembrane proteins embedded in glycolipid-containing bilayers. However, there continue to be significant challenges associated with experimental structural biology of such carbohydrate-containing systems. All-atom explicit-solvent molecular dynamics simulations provide a direct atomic resolution view of biomolecular dynamics and thermodynamics, but the accuracy of the results depends on the quality of the force field parametrization used in the simulations. A key determinant of the conformational properties of carbohydrates is ring puckering. Here, we applied extended system adaptive biasing force (eABF) all-atom explicit-solvent molecular dynamics simulations to characterize the ring puckering thermodynamics of the ten common pyranose monosaccharides found in vertebrate biology (as represented by the CHARMM carbohydrate force field). The results, along with those for idose, demonstrate that the CHARMM force field reliably models ring puckering across this diverse set of molecules, including accurately capturing the subtle balance between ^4^C_1_ and ^1^C_4_ chair conformations in the cases of iduronate and of idose. This suggests the broad applicability of the force field for accurate modeling of carbohydrate-containing vertebrate biomolecules such as glycoproteins, proteoglycans, and glycolipids.

## 1. Introduction

**Figure 1:**
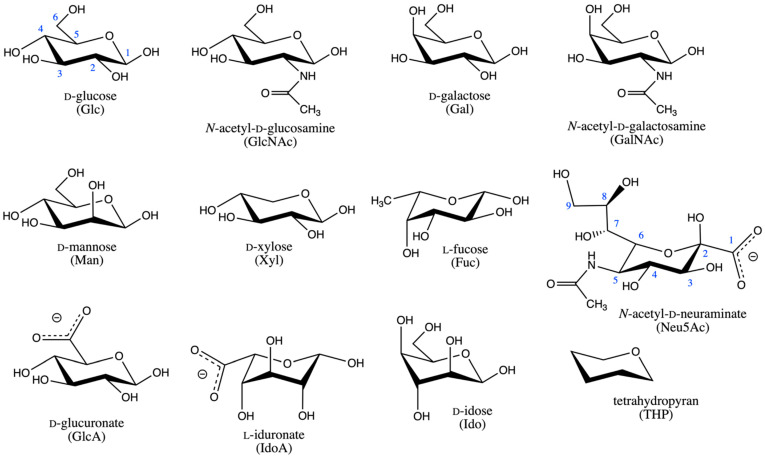
Compounds considered in the current study. Glc carbon atoms are numbered in blue. All other monosaccharides follow the same numbering scheme, except for Neu5Ac, which is numbered as pictured. All monosaccharides are drawn as the *β* anomer. The *α* anomer is created by inversion of the configuration at carbon 2 for Neu5Ac and at carbon 1 for all other monosaccharides. Both anomers for each monosaccharide as well as the corresponding O-methyl glycosides, formed by methylation at the anomeric carbon hydroxyl, were studied, for a total of 45 compounds (44 monosaccharides + THP).

Glycosylation is a common and important post-translational modification to proteins in eukaryotic biology. Additionally, carbohydrates are key components of eukaryotic lipids that make up the bilayers in which transmembrane proteins are embedded [1]. The carbohydrate portions of glycosylated proteins and glycolipids are called glycans. Naturally occurring glycans in vertebrates, including in humans, are composed of the monosaccharides D-glucose (Glc), *N*-acetyl-D-glucosamine (GlcNAc), D-galactose (Gal), *N*-acetyl-D-galactosamine (GalNAc), D-mannose (Man), D-xylose (Xyl), L-fucose (Fuc), *N*-acetyl-D-neuraminic acid (Neu5Ac), D-glucuronic acid (GlcA), and L-iduronic acid (IdoA), all in their pyranose forms [2] (Figure 1). As Neu5Ac, GlcA, and IdoA are expected to be deprotonated under typical physiological conditions, Figure 1 shows their conjugate base forms, *N*-acetyl-D-neuraminate, D-glucuronate, and L-iduronate, and it is these forms that are exclusively considered in what follows. Examples of glycans as components of glycosylated proteins are the *N*-glycans [3] and O-glycans [4] attached to glycoproteins and the glycosaminoglycans attached to proteoglycans [5]. Experimental atomic-resolution structural biology on glycosylated proteins is complicated by the non-template based synthesis of the attached glycans [6], which precludes a convenient source of homogeneous sample from biological sources, the intrinsic flexibility of glycans, which hinders conformational analysis by X-ray crystallography and NMR spectroscopy [7], and the covalent linkage of proteins with glycans, which can affect the structural properties of both the glycan and protein components [8,9,10]. In the context of membrane proteins, experimental atomic-resolution structural biology using X-ray crystallography entails extracting the membrane protein from its native lipid environment in order to create protein crystals [11], which means the effects of natural glycolipids in the native bilayer are not included in the structure determination. Therefore, the impact of glycans on protein structure continues to be at the frontiers of protein structure research.

**Figure 2:**
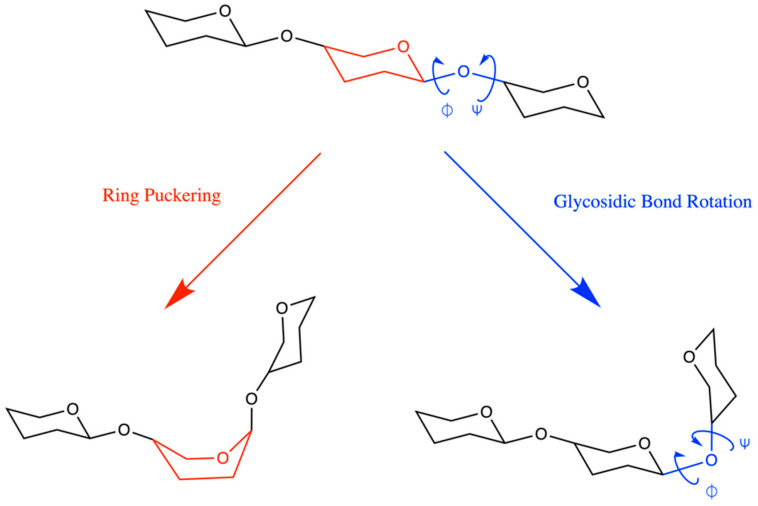
Pyranose ring puckering (red) and glycosidic bond rotation (blue) are the major sources of polymer flexibility in vertebrate glycans.

Computational approaches for three-dimensional modeling of the atomic-resolution conformational properties of glycans have been developed and applied to help bridge the gaps in experimental methods [12,13,14,15,16,17,18,19,20,21,22,23,24,25,26]. Widely used among these computational approaches are explicit solvent molecular dynamics simulations employing atomistic force fields such as GLYCAM06 [27,28], GROMOS 53A6GLYC [29,30], GROMOS 56a6CARBO/CARBO_R [31,32,33], OPLS-AA [34,35], and CHARMM [36,37,38,39]. The quality of the results from such molecular dynamics simulations depends upon the quality of the force field parametrization. The conformational properties of glycans are determined principally by flexibility in the rings of the constituent monosaccharides and in the glycosidic linkages connecting them (Figure 2) [12,40], and thus it is important for force field parametrizations to accurately capture the physics of these sources of flexibility in order to ensure reliable modeling results.

Since pyranose ring puckering occurs at the microsecond and beyond timescale [40,41,42], which is near the upper limit of typical present-day all-atom explicit-solvent molecular dynamics simulations, limitations in force field accuracy may not be readily apparent simply based on analysis of such simulation results. Here, we systematically determine the ring puckering thermodynamics of all compounds in Figure 2, including both the *α* and the *β* anomers and their corresponding O-methyl glycosides for the ten monosaccharides (i.e., 45 systems total), with the widely-used CHARMM force field. Extended System Adaptive Biasing Force (eABF) [43,44] is applied to achieve well-converged equilibrium statistics for ring puckering probabilities, with error estimates from triplicate 200-ns simulations for each system. The ring puckering thermodynamics from these simulations are in line with expected behavior, including for the highly flexible IdoA, and imply that the CHARMM force field can be used with confidence to correctly capture pyranose ring puckering contributions to glycan conformational heterogeneity in the context of the vertebrate glycans such as N-glycans, O-glycans, glycosaminoglycans, and glycolipids.

We additionally consider idose in its pyranose form, since, like IdoA, idose has a close balance between ^4^C_1_ and ^1^C_4_ chair probabilities, which makes it a useful test of force field accuracy. In agreement with prior computational results [45] and recent NMR data [46], the CHARMM carbohydrate force field performs very well in capturing the close balance for idose as well as for IdoA. Finally, for completeness, we include tetrahydropyran, which is the basic six-membered ring scaffold common to all of the monosaccharides considered here (Figure 2). As expected, there is an exact 50:50 balance for chair-chair interconversion for THP.

It is possible to tune ring puckering thermodynamics by selectively refining specific force field parameters and by using ring puckering thermodynamics as target data in the parametrization process. In the case of the GROMOS force field, such an approach was taken as a force field revision [31,32,47], and has yielded excellent results for ring puckering across a wide variety of pyranoses [32,33,45,48]. In the case of CHARMM, ring puckering thermodynamics in solution were not used as target data for CHARMM parametrization, and both bonded and nonbonded force field parameters, which built upon quantum mechanical gas phase puckering energetics for tetrahydropyran [36,49], are conserved across all of the different monosaccharides considered here. This demonstrates it is possible to correctly account for pyranose monosaccharide ring puckering thermodynamics in solution with a general transferable bonded and nonbonded force field parameter set. In the case of CHARMM, combining this parameter set with CHARMM force field parameters for proteins [50,51,52] can enable accurate modeling of glycoproteins and proteoglycans, and combining these parameters set with CHARMM force field parameters for lipids can do the same for glycolipids [53,54], which in turn can enable accurate modeling of transmembrane proteins embedded in complex bilayers composed of natural lipids.

## 2. Results and Discussion

### 2.1. Reaction Coordinate and Sampling Approach

Ring puckering for pyranose monosaccharides is commonly described using the Cremer-Pople (C-P) parameters (*Q*, *θ*, 𝜙) [55], which provide a convenient quantitative means to identify both the extent and the nature of the puckering using spherical coordinates. The puckering amplitude *Q* describes the extent or magnitude of the puckering, while the angular values 0° ≤ *θ* ≤ 180° and 0° ≤ 𝜙 < 360° describe the nature of the puckering. “Polar” values of *θ* near 0° and 180° correspond to ^4^C_1_ and ^1^C_4_ chair conformations, respectively, while “equatorial” values of *θ* near 90° correspond to boat and skew boat conformations, with the 𝜙 value indicating the specific boat or skew boat (e.g., ^2^S_O_). Intermediate or “tropical” values of *θ*, which are between the poles and the equator, correspond to envelope and half-envelope conformations, with the 𝜙 value indicating the specific envelope or half envelope [56].

Due to the long timescale for interconversion between ^4^C_1_ and ^1^C_4_, it is impractical to precisely determine the balance of probabilities and, hence, free energy difference, Δ*G*, between these conformations for pyranose monosaccharides on a routine basis using standard all-atom explicit-solvent molecular dynamics simulations. This is true for pyranoses where Δ*G* ≈ 0 due to the energy barrier separating the conformations [41,42], and the situation is even more difficult in cases where Δ*G* is substantially different than zero due to the difficulty in achieving equilibrium sampling of the unfavored conformation.

A logical means to address this issue is to apply a bias to *θ* during a simulation and either to reweight the sampling distribution to get unbiased conformational probabilities or to directly compute Δ*G* from the bias. Such an approach employing metadynamics [57,58] has enabled a number of studies to this end [29,32,33,45,48,59]. As demonstrated in these studies, this approach allows one to obtain a good estimate for Δ*G* with much less computation time than through standard (non-biased) molecular dynamics. There are two potential downsides to using a bias on *θ*. The first is the need to develop specialized computer code for the bias since the C-P *θ* is not a standard cartesian or internal coordinate. The second is that, while the single parameter *θ* can differentiate the two chair conformations from each other and also non-chair conformations from chair conformations, it cannot differentiate one non-chair conformation from another non-chair conformation. This second potential downside can be addressed by introducing a second simultaneous bias on 𝜙 but at the expense of further complicating the first downside.

For these reasons, direct use of dihedral angles is an attractive alternative. For example, Pickett and Strauss (P-S) defined three out-of-plane dihedrals constructed as various combinations of atoms in the pyranose ring [60], and it has been shown that simultaneous biases on all three of these angles can be effectively used to sample pyranose ring puckering [61]. In fact, the P-S and C-P approaches are mathematically equivalent [62]. However, there is an important practical difference with regard to applying biases on C-P parameters versus P-S out-of-plane dihedrals: only the two angular C-P parameters are required to uniquely identify the pucker nature (as opposed to magnitude) of a particular conformation whereas all three P-S out-of-plane dihedrals are required to do the same [59,63].

Babin and Sagui (B-S) have also proposed using dihedral angles for biased sampling of pyranose ring pucker [64]. In contrast to the P-S approach, only two dihedral angles are used in their scheme, α_1_ ≡ O5–C1–C2–C3 and α_2_ ≡ C3–C4–C5–O5, and the dihedral angles are real dihedrals determined by sequentially bonded atoms. Babin and Sagui have shown that biased sampling of (*α*_1_, *α*_2_) is an effective approach for sampling IdoA and GlcA puckering, and Alibay and Bryce have extended on these two monosaccharides to sulfated variants, as well as to non-sulfated and sulfated variants of GlcNAc, Gal, and GalNAc [65]. In what follows, we demonstrate that major minima in Δ*G*(α_1_, α_2_) are populated by unique conformations. As such, it is possible to do direct integration of regions of Δ*G*(α_1_, α_2_) to determine Δ*G* not only between the ^4^C_1_ and ^1^C_4_ chairs_,_ but also between specific boat/skew-boat conformations.

### 2.2. Extended System Adaptive Biasing Force (eABF) Sampling of the B-S (α_1_, α_2_) Reaction Coordinate

**Figure 3:**
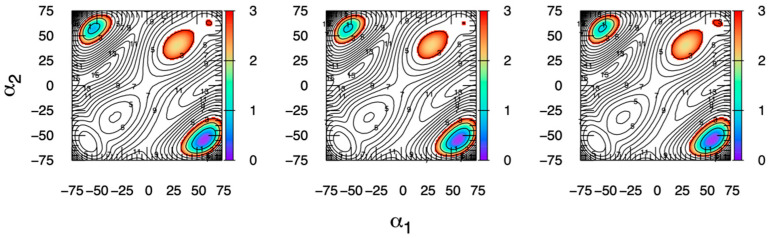
MeαIdoA Δ*G*(α_1_, α_2_) from eABF simulation. Each panel is from a separate 200-ns simulation seeded with different initial random velocities. α_1_ and α_2_ are in degrees. Δ*G*(α_1_, α_2_) is in kcal/mol, with contours drawn every 1 kcal/mol, colored from 0–3 kcal/mol, and labeled every 2 kcal/mol.

Methyl α-L-idopyranosiduronic acid (MeαIdoA) (Figure 2 “IdoA” with a methylated axial C1 hydroxyl) serves as a good test system to demonstrate the efficacy of eABF sampling of (α_1_, α_2_) owing to a small (<1 kcal/mol [46]) Δ*G* for conversion between the ^4^C_1_ and ^1^C_4_ chair conformations and a large energy barrier (~10 kcal/mol from the present work based on transition path saddle points in Figure 3), and therefore, slow kinetics, for this transition. Triplicate 200-ns eABF simulations with simultaneous biases on α_1_ and α_2_ and seeded with different randomized initial velocities yield essentially identical results across the entire Δ*G*(α_1_, α_2_) surface (Figure 3). Not only are the thermodynamic minima equal in both value and location, but so are the saddle regions and even the maxima, which demonstrates the excellent convergence properties of eABF for this system. Δ*G*(α_1_, α_2_) data are similarly well-converged for all 45 systems in this study (four different anomerization/methylation states for each of the 11 monosaccharides in Figure 2 plus tetrahydropyran; Supplementary Material Figures S1–S12).

**Figure 4:**
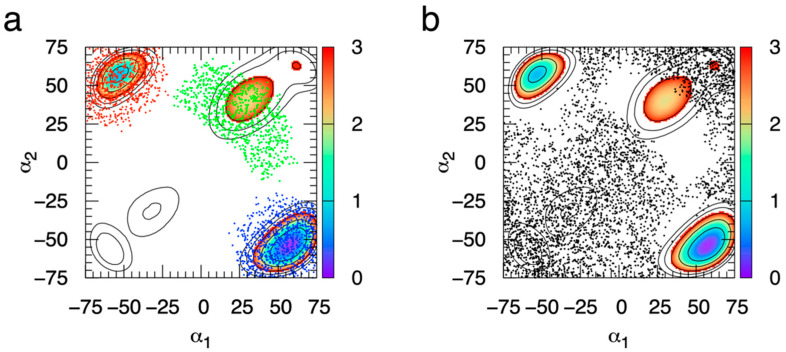
Sampling of specific MeαIdoA ring puckering conformations during eABF simulation with the Babin-Sagui (α_1_, α_2_) reaction coordinate. Sampled (α_1_, α_2_) values are separated into those for ^4^C_1_, ^1^C_4_, and ^2^S_O_ (blue, red, and green dots, respectively, in panel “a”) and for all other (black dots, panel “b”) puckering conformations. α_1_ and α_2_ are in degrees. Δ*G*(α_1_, α_2_) is in kcal/mol, with contours drawn every 1 kcal/mol from 0–5 kcal/mol and colored from 0–3 kcal/mol. Puckering data have been aggregated across the triplicate simulations, and Δ*G*(α_1_, α_2_) is from the first simulation in the triplicate.

Additionally, each major thermodynamic minimum, that is, where Δ*G*(α_1_, α_2_) < 3 kcal/mol, is populated by a single type of ring puckering conformation (Figure 4). We have chosen 3 kcal/mol as a cutoff value for the definition of major thermodynamic minimum since, at the simulation temperature of 298 K, values greater than 3 kcal/mol correspond to small probabilities, specifically, less than 0.64%. This association between a single ring puckering conformation and each major thermodynamic minimum in Δ*G*(α_1_, α_2_) holds for all 44 monosaccharides in this study, which illustrates the practical utility of the B-S reaction coordinate for characterizing pyranose ring puckering not only for chair conformations but also for specific non-chair conformations.

**Figure 5:**
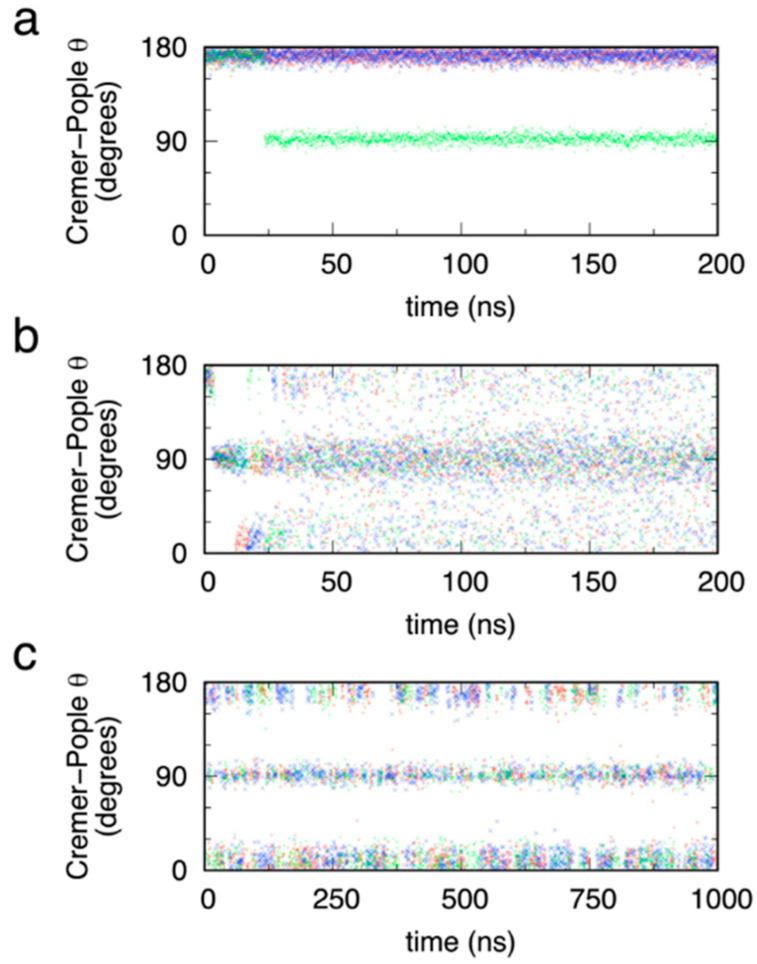
MeαIdoA conformational transitions in standard (non-biased) (**a**), eABF (**b**), and CMAP-biased (**c**) molecular dynamics simulations. eABF and CMAP biased simulations have biasing on the Babin-Sagui (α_1_, α_2_) reaction coordinate. Data in each panel are from triplicate simulations (blue, red, and green) seeded with different random initial velocities.

Kinetic data from the simulations clearly show the efficacy of eABF combined with the B-S (α_1_, α_2_) reaction coordinate for effectively sampling pyranose ring pucker, which is not surprising given the excellent convergence properties of Δ*G*(α_1_, α_2_) with eABF as discussed previously. Serving as a negative control, standard (non-biased) triplicate simulations of MeαIdoA starting from the ^1^C_4_ chair undergo at most one transition in C-P *θ* during 200 ns (Figure 5a). Specifically, two of the simulations maintain *θ* ≅ 180°, indicating they are trapped in the initial conformation, while the third transitions at 25 ns to *θ* ≅ 90° and remains there, indicating it is stuck in the equatorial boat/skew-boat region of puckering space. Therefore, standard sub-microsecond explicit solvent molecular dynamics simulation is inadequate for the task of sampling puckering conformations for pyranoses modeled with the CHARMM force field.

In contrast, with eABF sampling, during the first 25 ns, as the time-dependent biasing force becomes a progressively better estimate of the thermodynamic force along (α_1_, α_2_), transitions in *θ* start to become induced (Figure 5b). Beyond *t* = 25 ns, there is rapid transitioning on the nanosecond timescale from the ^4^C_1_ chair (*θ* ≅ 0°), through boat/skew-boat conformations (*θ* ≅ 90°), to the ^1^C_4_ chair (*θ* ≅ 90°) and back again, indicating sufficient sampling of (α_1_, α_2_) by eABF to provide an accurate estimate of the thermodynamic force along (α_1_, α_2_). As a technical point, the eABF approach applies a bias not to (α_1_, α_2_) directly but to extended degrees of freedom attached to (α_1_, α_2_), and the thermodynamic force on (α_1_, α_2_) is recovered from the biasing force applied to these extended degrees of freedom [43,44]. Standard ABF is not possible for sampling (α_1_, α_2_) since α_1_ and α_2_ do not meet the required orthogonality condition for standard ABF [66,67,68] owing to the sharing of atoms carbon 1 and oxygen 5 in both of the dihedral angle definitions. For additional information on this point, we refer interested readers to the cyclohexane data in Figure 2 of reference [44] and the associated discussion therein, which vividly demonstrates errors in estimation of cyclohexane puckering free energy with standard ABF that are corrected with eABF.

As a positive control, and similar to the approach of Babin and Sagui [64], we ran an additional set of simulations that employed CMAP-biased sampling [51,69]. In these simulations, the potential energy was defined by *U*_non-biased_ + *U*_CMAP_, where *U*_non-biased_ is the same CHARMM additive force field function used in the non-biased simulations here and *U*_CMAP_ is *U*_CMAP_(α_1_, α_2_) ≅ −0.5 × Δ*G*(α_1_, α_2_). Unlike in the eABF simulations, the bias, in this case from the CMAP term, is fixed. We note that *U*_CMAP_(α_1_, α_2_) is only approximately equal to −0.5 × Δ*G*(α_1_, α_2_) since, while Δ*G*(α_1_, α_2_) was computed on a square grid with a grid spacing of 1°, the grid spacing for the CMAP potential is 15°. As expected, there is excellent sampling of C-P *θ* from the very beginning of the triplicate simulations (Figure 5c). While there is rapid barrier crossing with this approach, there is less uniform sampling across all values of *θ* as compared to eABF sampling, with a strong tendency to favor sampling of polar and equatorial values of *θ* as compared to tropical values (Figure 5b vs. Figure 5c). This resulted from the factor of 0.5 used in the definition of *U*_CMAP_(α_1_, α_2_), and was done to maximize importance sampling of thermodynamically favored regions of (α_1_, α_2_) space while still lowering barriers sufficiently to achieve ergodic sampling of (α_1_, α_2_) on the 200-ns time scale of the simulations. As expected, thermodynamically unfavored regions of (α_1_, α_2_) correspond to tropical values, which in turn are envelope and half-envelope conformations with high degrees of ring strain.

**Figure 6:**
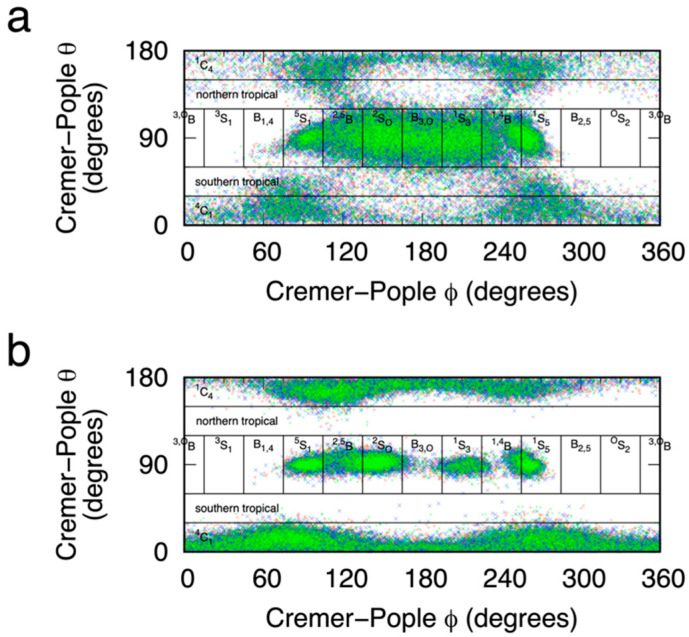
MeαIdoA Cremer-Pople (*θ*, 𝜙) values sampled during eABF (**a**) and CMAP-biased (**b**) molecular dynamics simulations. Pyranose ring puckering regions [56] (“^4^C_1_”, “northern tropical”, “^2^S_O_”, etc.) are labeled as defined in the Materials and Methods section. Biasing was applied to the Babin-Sagui (α_1_, α_2_) reaction coordinate. Data in each panel are from triplicate simulations (blue, red, and green) seeded with different random initial velocities. eABF simulations were 200 ns and CMAP-biased simulations were 1000 ns.

Plotting C-P (*θ*, 𝜙) values sampled during the eABF and the CMAP-biased simulations further validates the degree to which these two biasing methods applied to the (α_1_, α_2_) reaction coordinate enable sampling of pyranose puckering space. In addition to excellent coverage of the two chair conformations ^4^C_1_ and ^1^C_4_ located in the polar regions, there is good coverage of the equatorial region for 75° < 𝜙 < 270°, which includes ^5^S_1_, ^2,5^B, ^2^S_O_, B_3,O_, ^1^S_3_, ^1,4^B, and ^1^S_5_, in order of increasing 𝜙 (Figure 6). That said, there is very limited sampling of equatorial regions outside this range of 𝜙 values, resulting from the fact that the two-dimensional B-S (α_1_, α_2_) reaction coordinate is not a perfect replacement for biased sampling of the two-dimensional C-P (*θ*, 𝜙) reaction coordinate. Nonetheless, it is reasonable to assume conformations not sampled are very high in free energy and that the thermodynamically relevant conformations have all been sampled. This latter point is emphasized by comparing these sampling data for eABF versus CMAP biasing. In the case of eABF, as time increases, sampling approaches that for a distribution biased by −Δ*G*(α_1_, α_2_), whereas for CMAP biasing, sampling is that for a distribution biased by −0.5 × Δ*G*(α_1_, α_2_), as discussed above. As such, eABF provides more complete coverage of (*θ*, 𝜙) space (Figure 6a) as compared to CMAP-biased sampling (Figure 6b).

### 2.3. Using eABF-Computed ΔG(α_1_, α_2_) to Calculate Specific Ring Puckering Conformation Probabilities

Given that each major thermodynamic minimum for MeαIdoA is populated by a single type of puckering conformation, as shown above, it is possible simply to integrate the probabilities associated with each minimum to determine relative probabilities for specific ring puckering conformations. We operationalized this by converting Δ*G*(α_1_, α_2_) values from eABF simulations to probabilities *p*(α_1_, α_2_) using the Boltzmann relationship *p*(α_1_, α_2_) = exp(Δ*G*(α_1_, α_2_)/−*RT*), where *R* is the universal gas constant and *T* is the temperature. We then separated the data based on the (α_1_, α_2_) quadrant, and summed up all values of *p* for each (α_1_, α_2_) bin having an associated value Δ*G*(α_1_, α_2_) < 3 kcal/mol within a 20° degree radius of the most favorable thermodynamic minimum in that quadrant. This yields at most one summed probability, *P*, per quadrant of the (α_1_, α_2_) coordinate. In the case of MeαIdoA, there are three such values, *P*_+,+_, *P*_-,+_, and *P*_+,-_; the subscript here indicates the quadrant, for example, the quadrant defined by (α_1_ < 0°, α_2_ > 0°) for “−, +”. As discussed above, for MeαIdoA, the “+, −” minimum corresponds uniquely to the ^4^C_1_ ring pucker conformation, “−, +” to ^1^C_4_, and “+, +” to ^2^S_O_ (Figure 4), which allows for the assignment of probability values to specific ring pucker conformations based on eABF Δ*G*(α_1_, α_2_) results. 

### 2.4. Ring Puckering Probabilities: Idose and Iduronate

Among the molecules considered in this study (Figure 2), the Ido and IdoA compounds are well known to exhibit significant conformational flexibility with regard to ring pucker. There are recent high-quality experimental results quantifying this, but with variable agreement with prior molecular dynamics simulation studies [46]. Comparison of ^4^C_1_:^1^C_4_ ring puckering probability ratios shows good agreement between the present simulation results and these available experimental data (Table 1). In addition to probabilities from the eABF Δ*G*(α_1_, α_2_) results, we have included probabilities computed from the CMAP-biased simulations. These were determined by collecting all ^4^C_1_ conformations from a CMAP-biased simulation, assigning a probability *p* = exp(*U*_CMAP_/−*RT*) to each conformation to account for the effect of the CMAP bias, and then summing up the *p* values to get the total probability for the ^4^C_1_ pucker. This was likewise carried out for the ^1^C_4_ pucker, and the two total probabilities were normalized to sum to 100% (Table 1, “CMAP-biased simulations”).

**Figure 7:**
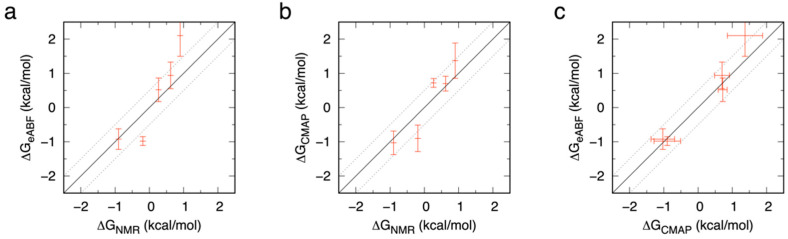
Comparison of Δ*G* values for the ^4^C_1_ to ^1^C_4_ equilibrium in Ido and IdoA compounds from eABF simulations, CMAP-biased simulations, and NMR experiments. Data are presented as eABF vs. NMR (**a**), CMAP-biased vs. NMR (**b**), and eABF vs. CMAP-biased (**c**). The specific compounds and the experimental data from NMR experiments are as detailed in Table 1. Simulation data points are averages from triplicate simulations, with error bars representing 95% confidence intervals. The solid diagonal is the line *y* = *x*, and the dotted diagonal lines are ±0.5 kcal/mol.

Converting the ^4^C_1_:^1^C_4_ ring puckering probability ratios *r* to free energies using the relationship Δ*G* = −*RT*ln(*r*) and plotting these Δ*G* values further illustrates how well the force field approach treats the close balance between ^4^C_1_ and ^1^C_4_ ring conformations. These Δ*G* values for the ^4^C_1_ to ^1^C_4_ equilibrium from the eABF and from the CMAP-biased simulations are typically within 0.5 kcal/mol of the experimental values (Figure 7a and Figure 7b, respectively). This very small degree of error is excellent for a force field model, and is not much different than what is seen when comparing the results from the two different simulation approaches using the same force field (Figure 7c).

### 2.5. Ring Puckering: ΔG(α_1_, α_2_) Minima for All Compounds

Quantitative calculation of pyranose ring puckering probabilities is valuable for comparison to high-quality experimental data for pyranoses with multiple thermodynamically accessible puckering conformations, as in the case of IdoA and Ido. However, such calculation by either integration around eABF Δ*G*(α_1_, α_2_) minima or summing of re-weighted probabilities for individual snapshots from CMAP-biased simulations entails substantial post-simulation effort following the initial computation of Δ*G*(α_1_, α_2_) with eABF. Unlike IdoA and Ido, most of the pyranose monosaccharides considered here are expected to have a single thermodynamically important Δ*G*(α_1_, α_2_) minimum that corresponds to either the ^4^C_1_ or ^1^C_4_ chair pucker conformation. As such, tabulation of Δ*G* minima values in the four quadrants of (α_1_, α_2_) space provides a convenient semi-quantitative means to evaluate the behavior of the force field model for those compounds.

Table 2 lists the Δ*G* minimum value in each of the four quadrants of (α_1_, α_2_) space for each of the 45 compounds studied. It also correlates each thermodynamically important minimum (i.e., having a value of <3 kcal/mol) with the puckering conformation associated with the value of (α_1_, α_2_) for that Δ*G* minimum. This correlation was carried out using computed Cremer-Pople parameters (detailed in “Materials and Methods: Definition of ^4^C_1_, ^1^C_4_, ^2^S_O_, ^O^S_2_, and other ring puckering conformations”) for trajectory snapshots with (α_1_, α_2_) values within a 10° radius of the location of the Δ*G* minimum.

As expected, most of the pyranose monosaccharides have a single major pucker conformation: the ^4^C_1_ or the ^1^C_4_ chair. Aside from IdoA and Ido, which were discussed in the previous section, exceptions to this are Me*β*GlcNAc, *β*GalNAc, Me*β*GalNAc, αXyl, αNeu5Ac, and MeαNeu5Ac.

Me*β*GlcNAc, *β*GalNAc, and Me*β*GalNAc all have their Δ*G*(α_1_, α_2_) global minimum corresponding to the ^4^C_1_ chair conformation, as expected. They each also have a secondary minimum, but in all three cases the associated Δ*G*(α_1_, α_2_) is no less than 2.5 kcal/mol, which translates to a probability of no more than 1.5%. For Me*β*GlcNAc, the secondary minimum arises from skew-boat puckering, whereas for *β*GalNAc and Me*β*GalNAc, the secondary minimum is the ^1^C_4_ chair conformation.

αXyl has the ^4^C_1_ chair conformation as its global minimum and a secondary Δ*G*(α_1_, α_2_) minimum corresponding to the ^1^C_4_ chair and with a value of 2.17 kcal/mol. This compares favorably to the value of 1.65 kcal/mol computed with the GROMOS 56a6CARBO force field (Table 1 in [48]), which is also exactly the value from Angyal’s scheme for determining ring puckering free energies [70]. We note that data from Angyal’s scheme have been used for quantitative comparison in other force field evaluations for a wide variety of pyranoses. It is worth emphasizing here that the Angyal data, though based in experiment, are indirect and were deemed by Angyal to be “calculated interaction energies”. Concerning his “calculated interaction energies”, Angyal writes, “an approximate calculation serves as a useful guide and can be readily carried out by adding the values of all of the non-bonded interactions occurring in each conformer, plus the value of the anomeric effect [70]”. That is, those Angyal data for the ^4^C_1_ to ^1^C_4_ equilibrium in D-aldopyranoses listed in Table 1 of [70] are calculated as a simple sum of experimental values from model compounds, in contrast to being directly measured for each monosaccharide, for example, through NMR experiments [46].

**Figure A1:**
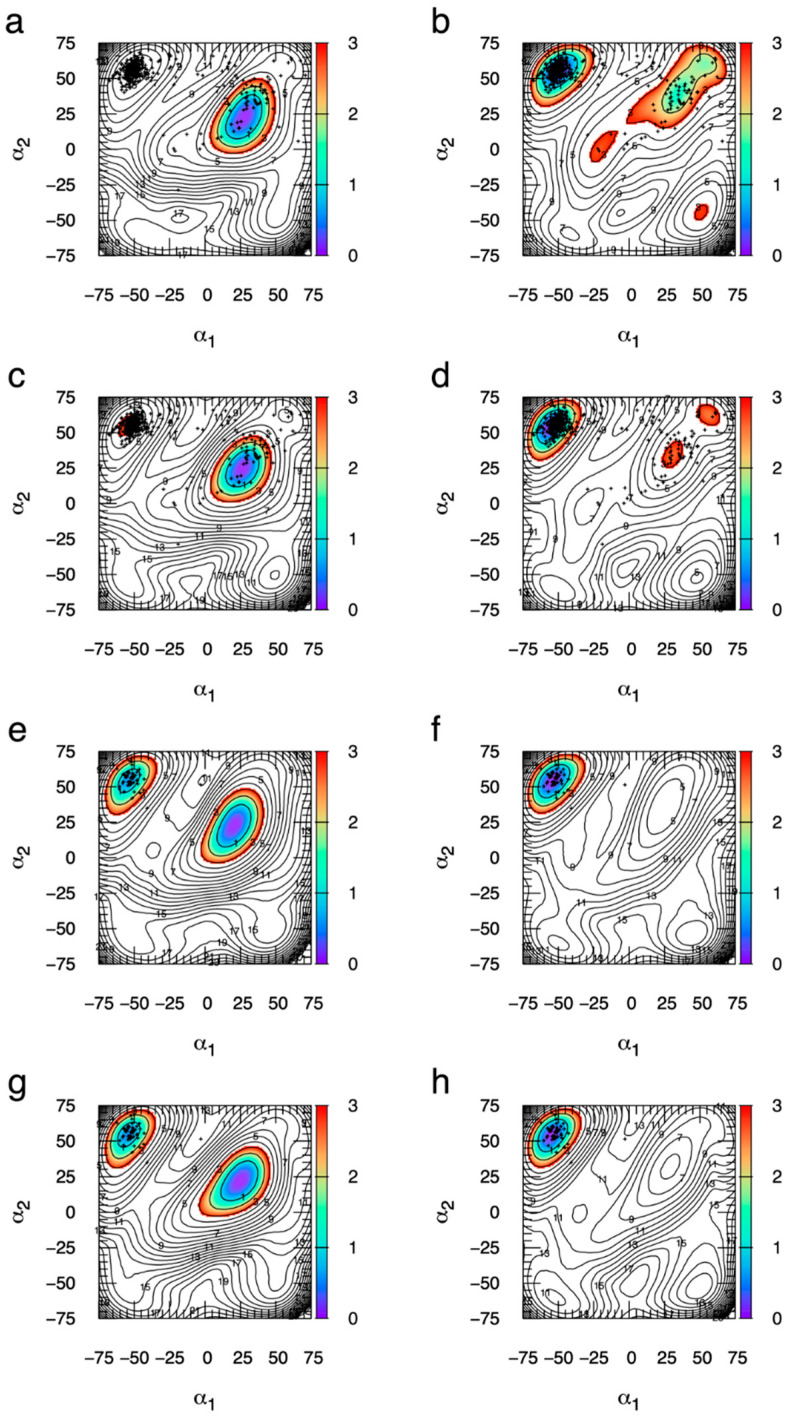
Δ*G*(α_1_, α_2_) from eABF simulations using incorrect versus correct dihedral force field parameters for Neu5Ac along with (α_1_, α_2_) data from all Neu5Ac structures in the PDB (searched 30 July 2021). Δ*G*(α_1_, α_2_) contour data are for αNeu5Ac with incorrect parameters (**a**), αNeu5Ac with correct parameters (**b**), MeαNeu5Ac with incorrect parameters (**c**), MeαNeu5Ac with correct parameters (**d**), *β*Neu5Ac with incorrect parameters (**e**), *β*Neu5Ac with correct parameters (**f**), Me*β*Neu5Ac with incorrect parameters (**g**), and Me*β*Neu5Ac with correct parameters (**h**). α_1_ and α_2_ are in degrees. Δ*G*(α_1_, α_2_) is from the first simulation in the triplicate and is in kcal/mol, with contours drawn every 1 kcal/mol and colored from 0–3 kcal/mol. PDB data were divided into two groups: those from α anomers and those from *β* anomers. Crystallographic data from the α anomers are displayed as small +’s in (**a**–**d**) and crystallographic data from the *β* anomers are displayed as small +’s in (**e**–**h**).

Neu5Ac is discussed at length in Appendix A, below. Part of that discussion involves comparison to structures from PDB crystal structures. On the one hand, all simulation data here are for isolated Neu5Ac monosaccharides in liquid water, whereas the PDB data are from crystal environments and typically involve Neu5Ac having non-covalent interactions with other biomolecules and/or being covalently attached to other monosaccharides. On the other hand, there is substantial congruence between the aqueous simulation data and the experimental crystal data for Neu5Ac (Figure A1b,d,f,h in Appendix A). Indeed, a computational study of Neu5Ac ring puckering in vacuum and in explicit water noted that the structure of Neu5Ac bound in influenza neuraminidase belonged to conformations preferentially sampled in the aqueous simulations [71]. And, an analysis of high-resolution PDB data for Me*β*GlcNAc noted that while nearly 97% of structures in the data set were in the ^4^C_1_ chair conformation, 2.6% were boats or skew boats [72], which correlates closely with data from the present work. Therefore, in addition to NMR data from directly analogous experimental systems of monosaccharides in liquid water, PDB data may be useful as benchmarks for the type of force field-based simulations described here.

On a final note, control eABF simulations for THP yield a Δ*G*(α_1_, α_2_) plot that is symmetric about both α_1_ = α_2_ and about α_1_ = −α_2_ (Supplementary Material Figure S12), as expected. There are two equivalent global minima at ^4^C_1_ = ^1^C_4_, and boat/skew-boat conformations are over 5 kcal/mol higher in free energy. Thus, the exocyclic functional groups in the pyranose monosaccharides considered here can be thought of as introducing two types of perturbations to the THP Δ*G*(α_1_, α_2_): breaking of the symmetry, and altering the balance of chair vs. boat/skew-boat energetics.

## 3. Materials and Methods

### 3.1. Force Field

All systems were modeled using the CHARMM all-atom additive force field for carbohydrates [36,38] and the CHARMM-modified TIP3P water parameters [73,74] as contained in the “jul20” release of the CHARMM force field available as “toppar_c36_jul20.tgz” from http://mackerell.umaryland.edu/charmm_ff.shtml (accessed on 3 March 2021). Systems with a carboxylate functional group additionally used sodium ion parameters [75,76] included in the same release. During the course of the present work, we discovered a set of typos in the jul20 parameter file that affect Neu5Ac puckering energetics. Full details are provided in Appendix A. The data presented in this manuscript and the associated Supplementary Material reflect the correct parameters as developed in [38]. 

### 3.2. System Construction

Solvated systems were constructed for each monosaccharide in Figure 2 using either the *α* or the *β* anomer or one of the corresponding O-methyl glycosides, resulting in four unique systems for each monosaccharide. Monosaccharide coordinates were constructed from default force field internal geometries. The solvent consisted of a cubic box of water molecules at the experimental density of water and having an edge length of the longest dimension of the monosaccharide plus 30 Å; water molecules within 3 Å of the monosaccharide were deleted. In systems with a carboxylate group, a single sodium ion replaced a water molecule randomly selected and at least 6 Å from the monosaccharide. All system construction was carried out using the CHARMM program, v. c45b1 [77]. A single system containing tetrahydropyran (THP) was similarly constructed.

### 3.3. Molecular Dynamics Simulations

Each system was simulated in triplicate under periodic boundary conditions. Each replicate within the triplicate was assigned random initial velocities using a unique random seed to generate a unique trajectory. Simulations were carried out using the NAMD software, v. 2.13 [78]. Electrostatic and Lennard-Jones interactions employed a 10-Å spherical cutoff. Lennard-Jones interaction energies were smoothly switched to zero in the interval 8–10 Å [79], an isotropic correction was applied for Lennard-Jones interactions beyond the cutoff [80], and the particle-mesh Ewald method with a 1 Å grid spacing accounted for electrostatic interactions beyond the cutoff [81]. After 1000 steps of energy minimization, each system was heated through re-initializing velocities to the target temperature of 298 K every 1000 molecular dynamics steps across 20,000 total steps with an integration timestep of 0.5 fs and positional restraints on solute non-hydrogen atoms. The SHAKE [82] and SETTLE algorithms [83] were respectively used to constrain all bonds involving hydrogen atoms and water geometries to their equilibrium values, and a temperature of 298 K and a pressure of 1 atm were maintained using Langevin thermostatting [84] and Nosé-Hoover Langevin barostatting [85,86]. Following heating, positional restraints were removed and data were collected from 200-ns production simulations (100 × 10^6^ timesteps with an integration timestep of 2.0 fs).

The Extended-System Adaptive Biasing Force (eABF) methodology [43,44] was used to determine the free energy of pyranose ring puckering, Δ*G*(*α*_1_, *α*_2_), using reaction coordinates proposed by Babin and Sagui [64], where *α*_1_ is the dihedral angle defined by the atoms O5-C1-C2-C3 and *α*_2_ is the dihedral defined by the atoms C3-C4-C5-O5, except in the case of sialic acid in which these dihedrals are defined by O6-C2-C3-C4 and C4-C5-C6-O6, respectively. Δ*G*(*α*_1_, *α*_2_) was computed from the CZAR gradient estimate [43] using a Poisson equation formalism [87] implemented within NAMD via the Colvars software module [88]. eABF parameters included a fictitious particle spring constant of *k*_B_*T*/degree/degree and sampling with a 1° × 1° bin size and restrained with half-harmonic potentials to the range −75° < *α*_1,2_ < 75°. Application of the biasing force in a given bin was scaled by 0 for the first 100 samples and then linearly scaled from 0 to 100% between 100 and 200 samples. Non-biased control simulations followed the same protocol but with no eABF sampling.

Additional CMAP-biased simulations were carried out for iduronate and for idose by applying a fixed bias equal to −0.5 × Δ*G*(*α*_1_, *α*_2_) through the CHARMM force field CMAP term [69]. The representation of this bias using CMAP is not exact relative to the reference values computed by eABF simulation, as CMAP uses a square grid with 15° intervals between grid points and bicubic interpolation approximate −0.5 × Δ*G*(*α*_1_, *α*_2_) for off-grid values of (*α*_1_, *α*_2_). CMAP-biased simulations were run using the OpenMM software, v. 7.5.1 [89] and a molecular dynamics protocol similar to that used for non-biased control NAMD simulations.

### 3.4. Molecular Dynamics Trajectory Analysis

Molecular dynamics trajectories were analyzed with the CHARMM software, including for the computation of Cremer-Pople ring puckering parameters [55]. VMD [90] was used for visualization and the creation of molecular graphics.

### 3.5. Definition of ^4^C_1_, ^1^C_4_, ^2^S_O_, ^O^S_2_, and Other Ring Puckering Conformations

C-P parameters (*θ*, 𝜙) were used to define ring puckering conformations as follows (note: analogous puckers for Neu5Ac compounds have all superscripted/subscripted numbers in puckering conformations incremented by 1 to reflect the different atom numbering in Neu5Ac, as shown in Figure 2):

- ^4^C_1_: 0° ≤ *θ* < 30°, 𝜙 = any
- Southern tropical: 30° ≤ *θ* < 60°, 𝜙 = any
- Equatorial: 60° ≤ *θ* < 120°, with specific conformations defined by,
  ○ ^3,O^B: 0° ≤ 𝜙 < 15° or 345° ≤ 𝜙 < 360°
  ○ ^3^S_1_: 15° ≤ 𝜙 < 45°
  ○ B_1,4_: 45° ≤ 𝜙 < 75°
  ○ ^5^S_1_: 75° ≤ 𝜙 < 105°
  ○ ^2,5^B: 105° ≤ 𝜙 < 135°
  ○ ^2^SO: 135° ≤ 𝜙 < 165°
  ○ B_3,O_: 165° ≤ 𝜙 < 195°
  ○ ^1^S_3_: 195° ≤ 𝜙 < 225°
  ○ ^1,4^B: 225° ≤ 𝜙 < 255°
  ○ ^1^S_5_: 255° ≤ 𝜙 < 285°
  ○ B_2,5_: 285° ≤ 𝜙 < 315°
  ○ ^O^S_2_: 315° ≤ 𝜙 < 345°
- Northern tropical: 120° ≤ *θ* < 150°, 𝜙 = any
- ^4^C_1_: 150° ≤ *θ* ≤ 180°, 𝜙 = any

## 4. Conclusions

The data presented here provide a thorough accounting of the ring puckering free energies for the ten common vertebrate monosaccharides and idose, as represented by the CHARMM force field. In addition to demonstrating that the CHARMM force field reliably models ring puckering across this set diverse of molecules, the results show that doing so is possible with a single set of self-consistent force-field parameters developed using a standardized force field parametrization protocol [36,38]. This, in combination with examples of CHARMM force field studies on glycosidic linkages [91,92,93,94,95,96], lends confidence to the application of these parameters in the modeling of carbohydrate-containing protein systems, such as glycoproteins and proteoglycans as well as transmembrane proteins in glycolipid-containing bilayers. Accurate simulations for these types of systems can help expand the frontiers of protein structural biology by bridging gaps in experimental approaches for characterizing carbohydrate-containing protein systems.

## Figures and Tables

**Table 1:** ^4^C_1_:^1^C_4_ ring puckering probability ratios in idose (Ido) and iduronate (IdoA) compounds.

Compound	eABF Simulations ^1^	CMAP-Biased Simulations ^1^	Experimental [46]
αIdo	17.6:82.4 (1.8)	15.1:84.9 (1.9)	18:82
MeαIdo	16.1:83.9 (0.7)	18.1:81.9 (2.1)	42:58
*β*Ido	97.1:2.9 (0.7)	90.7:9.3 (1.7)	82:18
Me*β*Ido	82.8:17.2 (2.2)	76.6:23.4 (1.5)	74:26
MeαIdoA	82.9:17.1 (1.4)	77.2:22.8 (1.0)	61:39

^1^ Data are averages from triplicate simulations with standard error of the mean values in parentheses.

**Table 2:** Minimum Δ*G*(α_1_, α_2_) values in each of the four quadrants of the (α_1_, α_2_) reaction coordinate, and the corresponding major ring puckering conformation(s).

Compound	Δ*G*_+,−_^1^	Δ*G*_−,+_^1^	Δ*G*_−,−_^1^	Δ*G*_+,+_^1^	Major Pucker Conformation(s) ^2^
αGlc	0	5.47 (0.04)	6.05 (0.04)	8.46 (0.05)	^4^C_1_
MeαGlc	0	6.83 (0.06)	7.06 (0.05)	9.39 (0.06)	^4^C_1_
*β*Glc	0	8.43 (0.19)	5.44 (0.04)	7.03 (0.01)	^4^C_1_
Me*β*Glc	0	8.27 (0.08)	5.38 (0.03)	6.91 (0.02)	^4^C_1_
αGlcNAc	0	5.01 (0.05)	6.11 (0.07)	7.19 (0.05)	^4^C_1_
MeαGlcNAc	0	6.19 (0.11)	7.08 (0.04)	8.20 (0.04)	^4^C_1_
*β*GlcNAc	0	4.95 (0.09)	4.01 (0.02)	6.60 (0.05)	^4^C_1_
Me*β*GlcNAc	0	4.73 (0.06)	2.83 (0.09)	6.85 (0.04)	^4^C_1_ > ^1^S_5_
αGal	0	4.25 (0.06)	6.11 (0.04)	8.73 (0.06)	^4^C_1_
MeαGal	0	5.62 (0.01)	7.35 (0.04)	8.74 (0.03)	^4^C_1_
*β*Gal	0	6.56 (0.09)	5.80 (0.01)	8.35 (0.04)	^4^C_1_
Me*β*Gal	0	7.10 (0.06)	6.63 (0.04)	8.29 (0.04)	^4^C_1_
αGalNAc	0	3.09 (0.09)	7.00 (0.07)	7.72 (0.09)	^4^C_1_
MeαGalNAc	0	4.33 (0.06)	8.21 (0.05)	7.77 (0.06)	^4^C_1_
*β*GalNAc	0	2.47 (0.05)	3.66 (0.07)	7.03 (0.04)	^4^C_1_ > ^1^C_4_
Me*β*GalNAc	0	2.90 (0.04)	3.58 (0.01)	6.87 (0.05)	^4^C_1_ > ^1^C_4_
αMan	0	5.26 (0.6)	6.83 (0.02)	9.99 (0.06)	^4^C_1_
MeαMan	0	5.82 (0.03)	7.54 (0.06)	10.74 (0.03)	^4^C_1_
*β*Man	0	6.89 (0.05)	7.27 (0.03)	8.98 (0.04)	^4^C_1_
Me*β*Man	0	6.20 (0.05)	6.24 (0.05)	8.14 (0.01)	^4^C_1_
αXyl	0	2.17 (0.01)	6.03 (0.02)	6.00 (0.05)	^4^C_1_ > ^1^C_4_
MeαXyl	0	3.60 (0.02)	6.95 (0.02)	6.73 (0.02)	^4^C_1_
*β*Xyl	0	3.77 (0.05)	5.25 (0.01)	3.24 0.01)	^4^C_1_
Me*β*Xyl	0	4.19 (0.00)	4.91 (0.02)	3.51 (0.01)	^4^C_1_
αFuc	3.87 (0.06)	0	8.11 (0.06)	5.97 (0.02)	^1^C_4_
MeαFuc	5.14 (0.03)	0	8.15 (0.01)	7.10 (0.02)	^1^C_4_
*β*Fuc	6.48 (0.03)	0	8.10 (0.03)	5.73 (0.01)	^1^C_4_
Me*β*Fuc	7.01 (0.04)	0	7.86 (0.02)	6.54 (0.04)	^1^C_4_
αNeu5Ac	2.71 (0.09)	0	2.79 (0.02)	1.42 (0.07)	^2^C_5_ > ^3^S_O_ > ^5^C_2_ ≅ ^4,O^B
MeαNeu5Ac	4.89 (0.29)	0	6.37 (0.10)	2.71 (0.24)	^2^C_5_ > ^3^S_O_
*β*Neu5Ac	6.79 (0.09)	0	7.18 (0.10)	4.01 (0.03)	^1^C_4_
Me*β*Neu5Ac	8.76 (0.08)	0	8.88 (0.10)	5.93 (0.11)	^1^C_4_
αGlcA	0	4.53 (0.12)	5.64 (0.04)	6.28 (0.05)	^4^C_1_
MeαGlcA	0	5.80 (0.04)	6.75 (0.03)	7.20 (0.03)	^4^C_1_
*β*GlcA	0	5.96 (0.11)	5.69 (0.01)	4.31 (0.06)	^4^C_1_
Me*β*GlcA	0	8.30 (0.09)	6.49 (0.02)	6.22 (0.04)	^4^C_1_
αIdoA	0	0.31 (0.09)	3.84 (0.04)	1.74 (0.02)	^4^C_1_ ≅ ^1^C_4_ > ^2^S_O_
MeαIdoA	0	0.77 (0.05)	3.23 (0.01)	2.04 (0.02)	^4^C_1_ > ^1^C_4_ > ^2^S_O_
*β*IdoA	2.29 (0.03)	0	4.47 (0.08)	3.81 (0.03)	^1^C_4_ > ^4^C_1_
Me*β*IdoA	3.29 (0.06)	0	4.31 (0.05)	3.53 (0.06)	^1^C_4_
αIdo	0.73 (0.08)	0	0.88 (0.08)	3.20 (0.06)	^1^C_4_ > ^4^C_1_ ≅ ^O^S_2_
MeαIdo	0.82 (0.04)	0	1.00 (0.02)	2.81 (0.03)	^1^C_4_ > ^4^C_1_ ≅ ^O^S_2_ > ^3^S_1_
*β*Ido	0	2.15 (0.10)	4.17 (0.08)	5.30 (0.09)	^4^C_1_ > ^1^C_4_
Me*β*Ido	0	1.12 (0.09)	3.49 (0.04)	5.00 (0.09)	^4^C_1_ > ^1^C_4_
THP	0	0.03 (0.01)	5.14 (0.01)	5.13 (0.00)	^4^C_1_ = ^1^C_4_

^1^ Data in kcal/mol are averages from triplicate simulations for the minimum Δ*G*(α_1_, α_2_) in that quadrant. For example, “**_−_**,+” indicates the quadrant defined by (α_1_ < 0°, α_2_ > 0°). Standard error of the mean values are in parentheses. ^2^ Conformations are listed in order of most likely to least likely. Only conformations corresponding to Δ*G*_+,−_, Δ*G*_−,+_, Δ*G*_+,+_, and/or Δ*G*_−,−_ < 3 kcal/mol are listed.

## Supplementary Materials

The following are available online at www.mdpi.com/article/10.3390/ijms23010473/s1.

## Appendix A

During the course of the present work, we discovered a set of typos in the jul20 parameter file that affect Neu5Ac puckering energetics. These typos affect only Neu5Ac in the present work and will be corrected in a future official update to the CHARMM force field (A. D. MacKerell, Jr., personal communication). For the time being, the jul20 “par_all36_carb.prm” CHARMM parameter file can be manually corrected by adding the following lines to that file and deleting all other lines that refer to these same parameters:

NC2D1	CC3161	CC3161	CC3261	0.20	3	0.0
CC312	CC3163	CC3161	NC2D1	0.20	3	0.0
OC3C61	CC3163	CC3161	NC2D1	0.20	3	0.0

The typos affect two dihedrals in the Neu5Ac ring, with the first parameter affecting rotation about the C4-C5 bond and the second two rotation about the C5-C6 bond. The above three lines revert the parameters to the original values in the publication describing parametrization for Neu5Ac [38]. Figure A1 demonstrates the large qualitative difference between the eABF Δ*G*(α_1_, α_2_) results using the incorrect force field dihedral parameters resulting from the typos and the correct force field dihedral parameters that are the original values from that publication. In Figure A1, (α_1_, α_2_) values from all instances of Neu5Ac in the PDB are overlaid on top of the Δ*G*(α_1_, α_2_) contour plots, and clearly show the superiority of the correct force field parameters as judged by the overlap of the PDB data with the global minima in the Δ*G*(α_1_, α_2_) contour plots (data were extracted from the PDB on 30 July 2021 by searching with the SMILES string “CC(=O)NC1C(CC(OC1C(C(CO)O)O)(C(=O)O)O)O” and separating hits into either α anomers or *β* anomers, of which there were 439 and 52, respectively found across a total of 170 PDB entries). In the case of the incorrect parameters, there is poor overlap, while with the correct parameters there is excellent overlap.

For αNeu5Ac and MeαNeu5Ac simulated using the incorrect parameters (Figure A1a and Figure A1c, respectively), the global minimum is in a boat/skew-boat region of (α_1_, α_2_) space whereas the vast majority of crystallographic structures in the α anomeric form are in the ^2^C_5_ chair pucker conformation. However, with the correct force field parameters, the global minimum is in the ^2^C_5_ region of (α_1_, α_2_) space for both αNeu5Ac (Figure A1b) and MeαNeu5Ac (Figure A1d), and the small proportion of α anomeric crystallographic structures outside of this region are located in or near a secondary minimum with favorable free energy (i.e., < 3 kcal/mol).

For the *β* anomers simulated using the incorrect parameters (*β*Neu5Ac (Figure A1e) and Me*β*Neu5Ac (Figure A1g)), there are no crystallographic Neu5Ac structures in the *β* anomeric form that coincide with the global minimum. In contrast, with the correct parameters, nearly all of these crystallographic structures in the *β* anomeric form, which are in the ^2^C_5_ chair pucker conformation, coincide with the global Δ*G*(α_1_, α_2_) minimum for both *β*Neu5Ac (Figure A1f) and Me*β*Neu5Ac (Figure A1h).
