# Supplementary material for: Pyranose Ring Puckering Thermodynamics for Glycan Monosaccharides Associated with Vertebrate Proteins
Source: Int J Mol Sci. 2021 Dec 31;23(1):473. doi: 10.3390/ijms23010473 (PMC8745717; doi:10.3390/ijms23010473)
Supplement: Supplementary file 1 [file ijms-23-00473-s001.zip › ijms-1520682-supplementary.pdf]

Supplementary Material for:

Pyranose ring puckering thermodynamics for common vertebrate monosaccharides and idose with the CHARMM force field

Olgun Guvench,[1,2,*] Devon Martin,[1,2] and Megan Greene[1]

[1] Department of Pharmaceutical Sciences and Administration, University of New England School of Pharmacy, 716 Stevens Avenue, Portland, Maine, 04103, USA; dmartin11@une.edu (D.M.); mgreene3@une.edu (M.G.)

[2] Graduate School of Biomedical Science and Engineering, University of Maine, 5775 Stodder Hall, Orono, Maine, 04469, USA

[*] Correspondence: oguvench@une.edu; Tel.: +01-207-221-4171

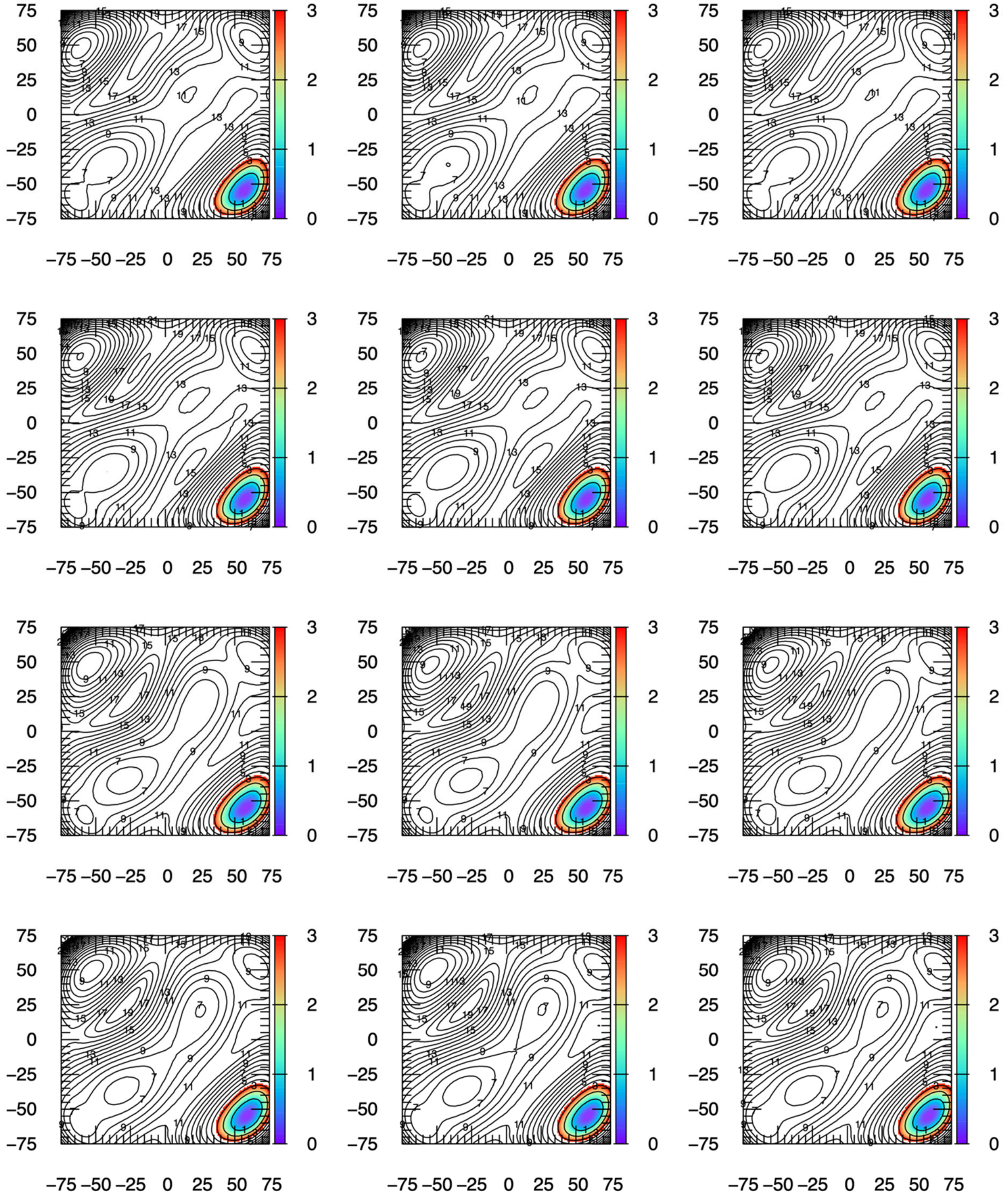

Figure S1. Triplicate eABF $\Delta G(\alpha_1, \alpha_2)$ data for the α- and β-anomers of D-glucose (Glc) and the corresponding O-methyl glycosides. First row: αGlc; second row: MeαGlc; third row: βGlc; fourth row: MeβGlc. $\Delta G(\alpha_1, \alpha_2)$ is in kcal/mol, with contours drawn every 1 kcal/mol, colored from 0-3 kcal/mol, and labeled every 2 kcal/mol.

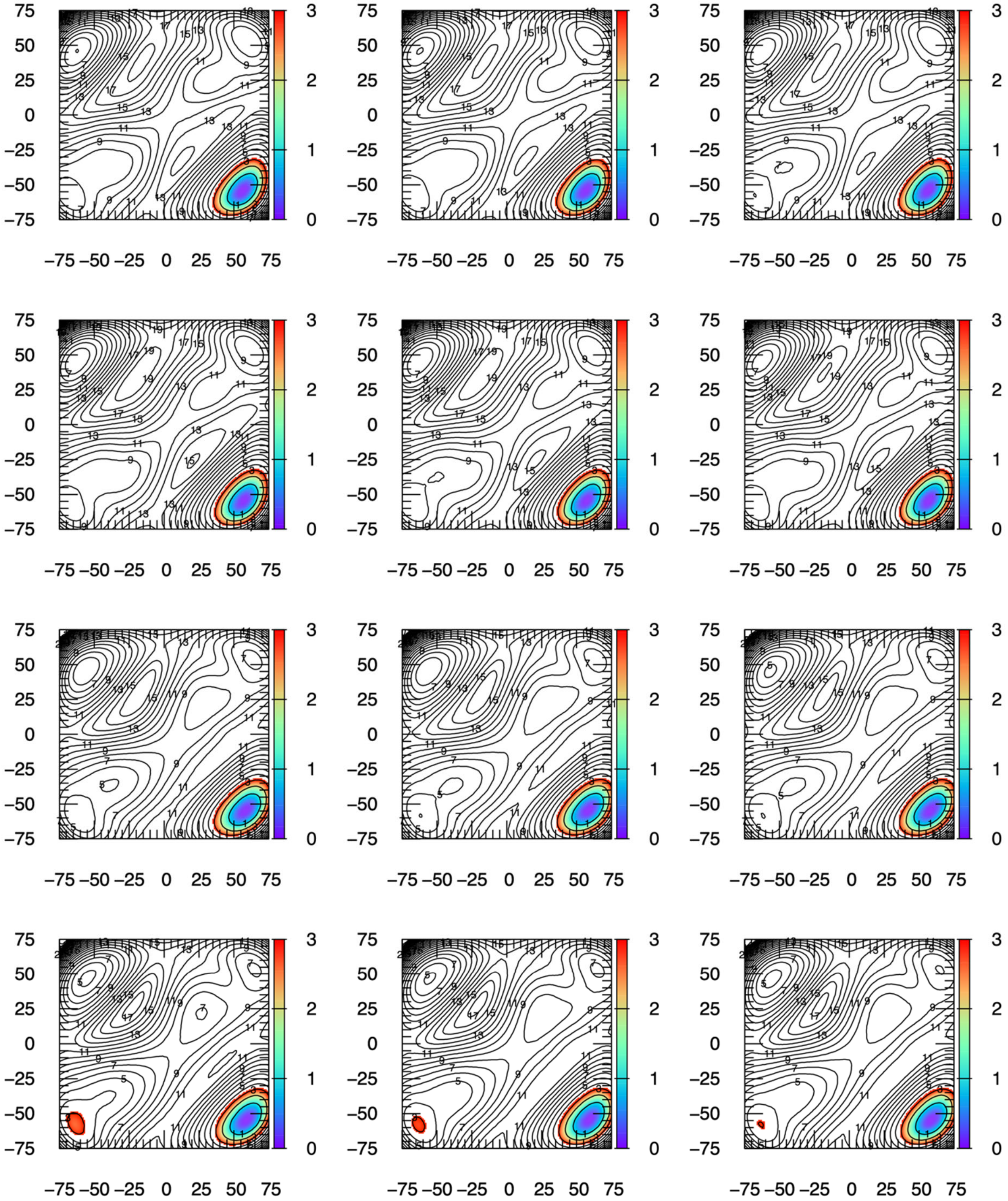

Figure S2. Triplicate eABF $\Delta G(\alpha_1, \alpha_2)$ data for the α- and β-anomers of *N*-acetyl-D-glucosamine (GlcNAc) and the corresponding O-methyl glycosides. First row: αGlcNAc; second row: MeαGlcNAc; third row: βGlcNAc; fourth row: MeβGlcNAc. $\Delta G(\alpha_1, \alpha_2)$ is in kcal/mol, with contours drawn every 1 kcal/mol, colored from 0-3 kcal/mol, and labeled every 2 kcal/mol.

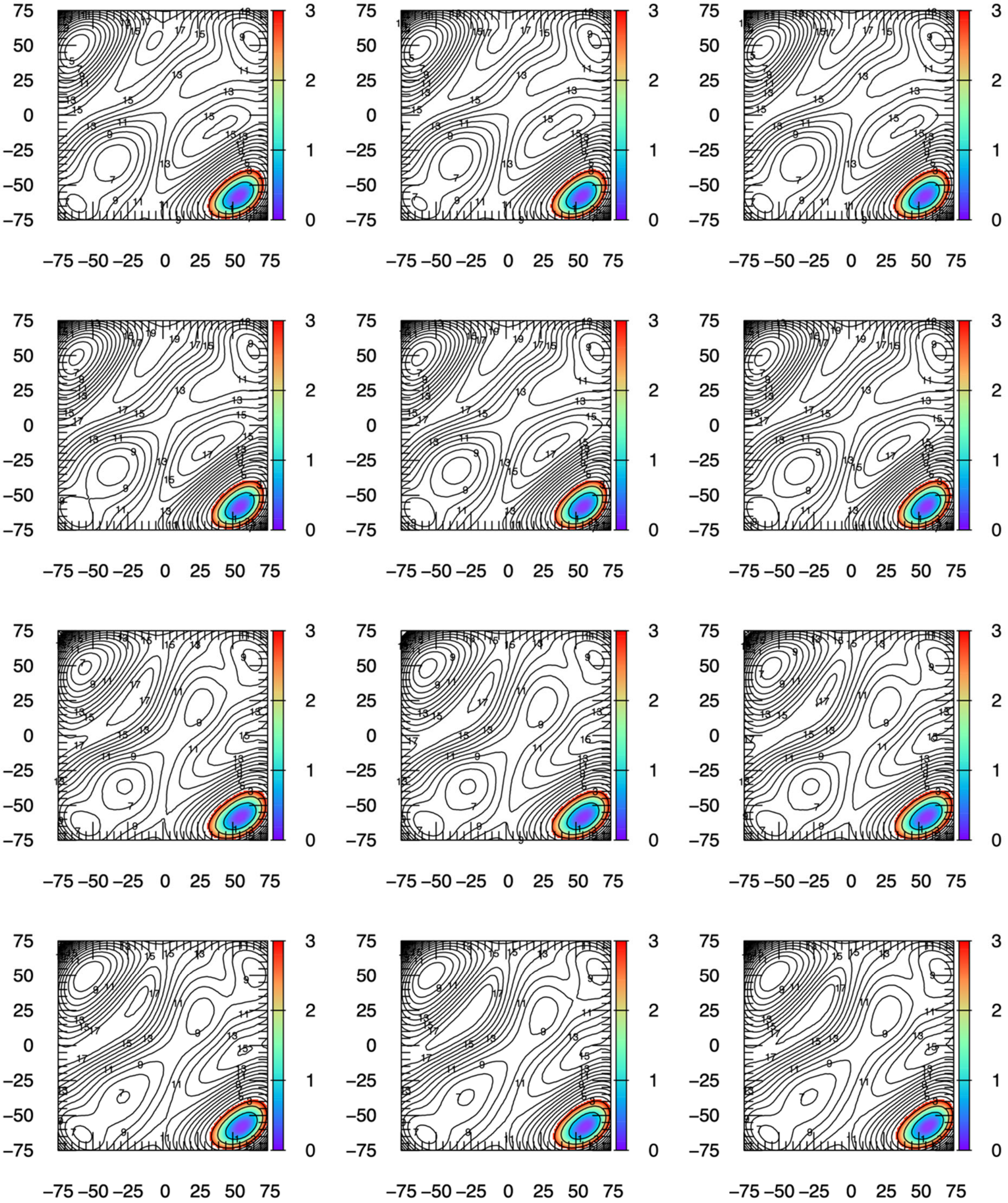

Figure S3. Triplicate eABF $\Delta G(\alpha_1, \alpha_2)$ data for the α- and β-anomers of D-galactose (Gal) and the corresponding O-methyl glycosides. First row: αGal; second row: MeαGal; third row: βGal; fourth row: MeβGal. $\Delta G(\alpha_1, \alpha_2)$ is in kcal/mol, with contours drawn every 1 kcal/mol, colored from 0-3 kcal/mol, and labeled every 2 kcal/mol.

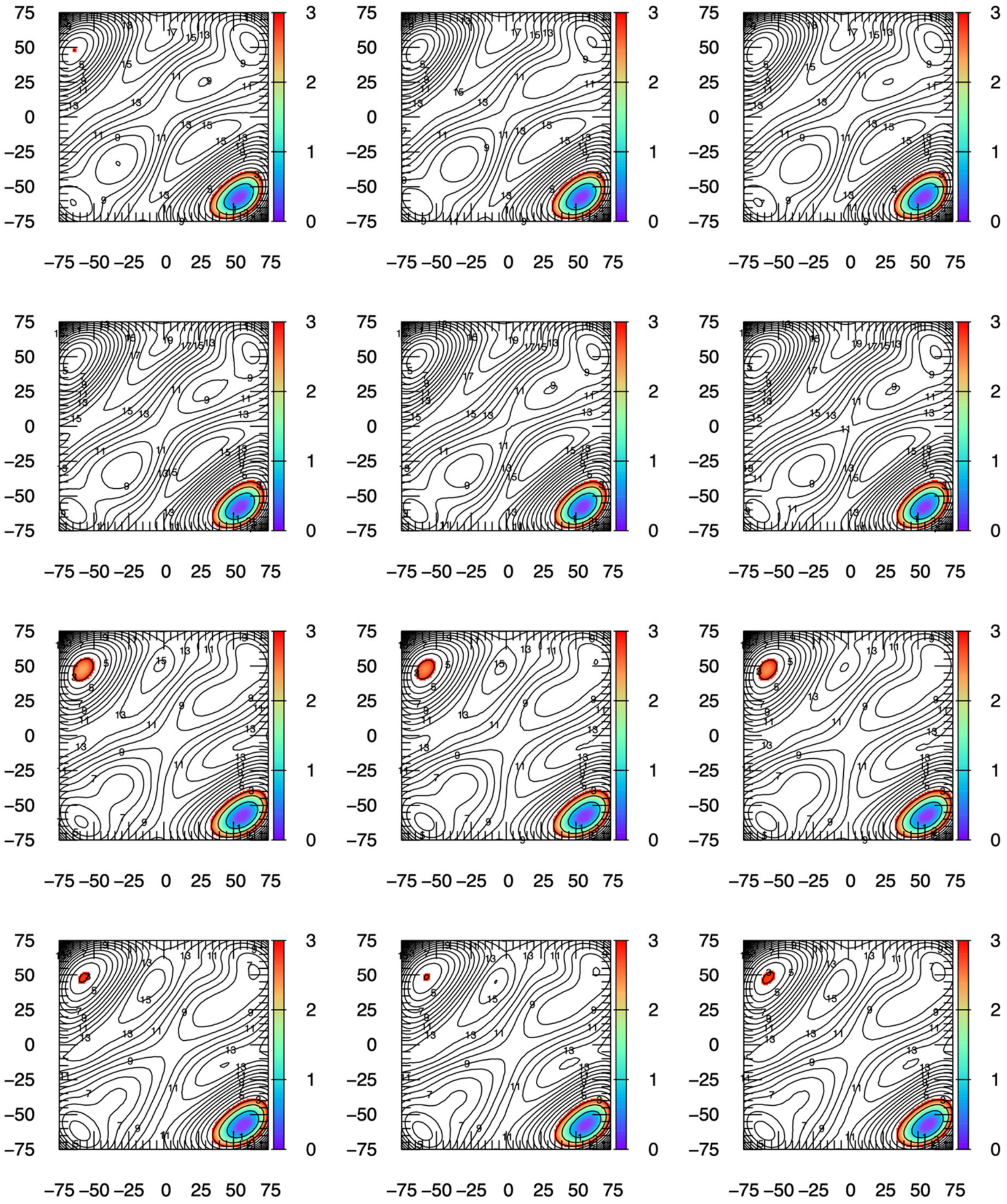

Figure S4. Triplicate eABF $\Delta G(\alpha_1, \alpha_2)$ data for the α- and β-anomers of *N*-acetyl-D-galactosamine (GalNAc) and the corresponding O-methyl glycosides. First row: αGalNAc; second row: MeαGalNAc; third row: βGalNAc; fourth row: MeβGalNAc. $\Delta G(\alpha_1, \alpha_2)$ is in kcal/mol, with contours drawn every 1 kcal/mol, colored from 0-3 kcal/mol, and labeled every 2 kcal/mol.

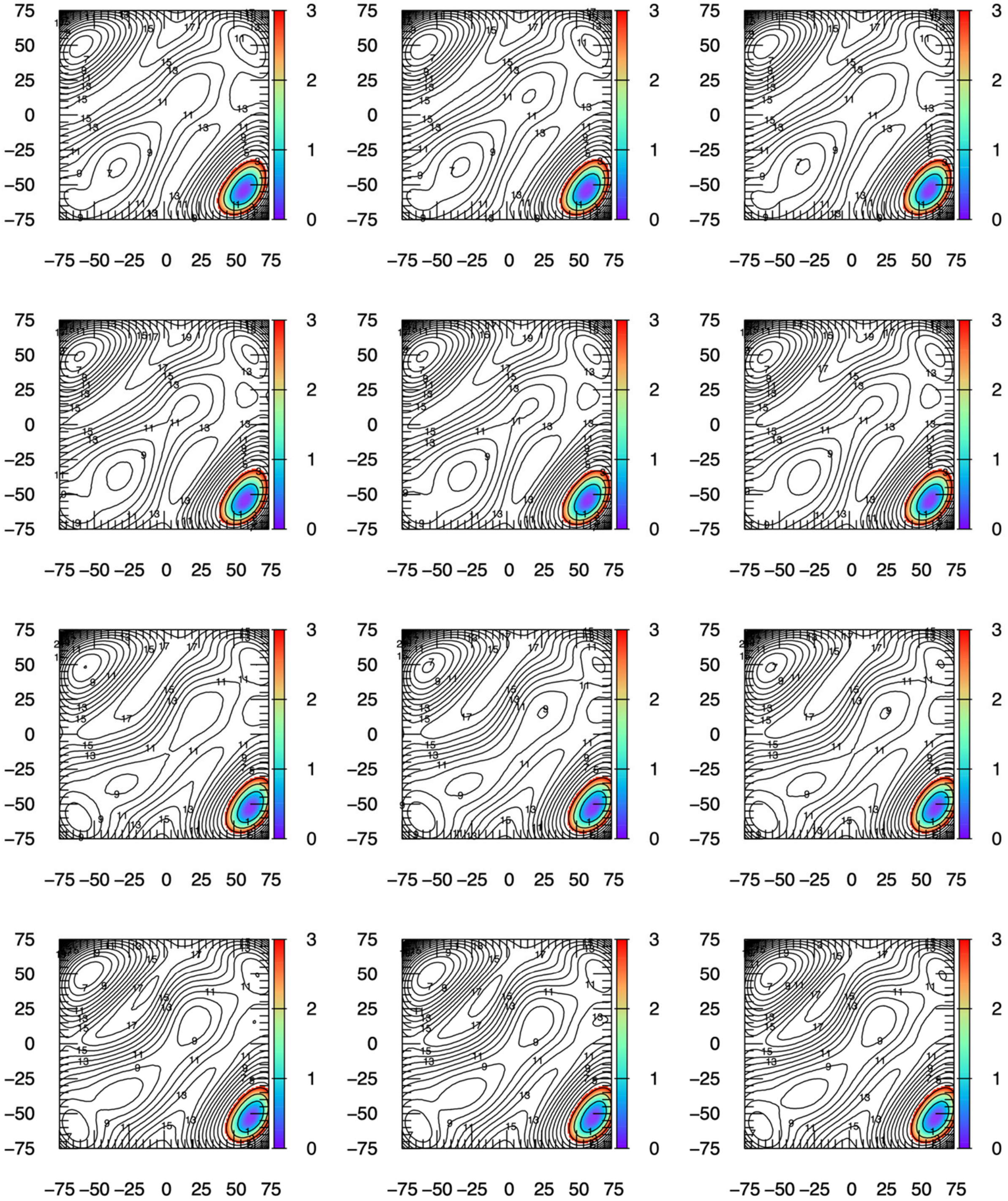

Figure S5. Triplicate eABF $\Delta G(\alpha_1, \alpha_2)$ data for the *α*- and *β*-anomers of D-mannose (Man) and the corresponding O-methyl glycosides. First row: *α*Man; second row: Me*α*Man; third row: *β*Man; fourth row: Me*β*Man. $\Delta G(\alpha_1, \alpha_2)$ is in kcal/mol, with contours drawn every 1 kcal/mol, colored from 0-3 kcal/mol, and labeled every 2 kcal/mol.

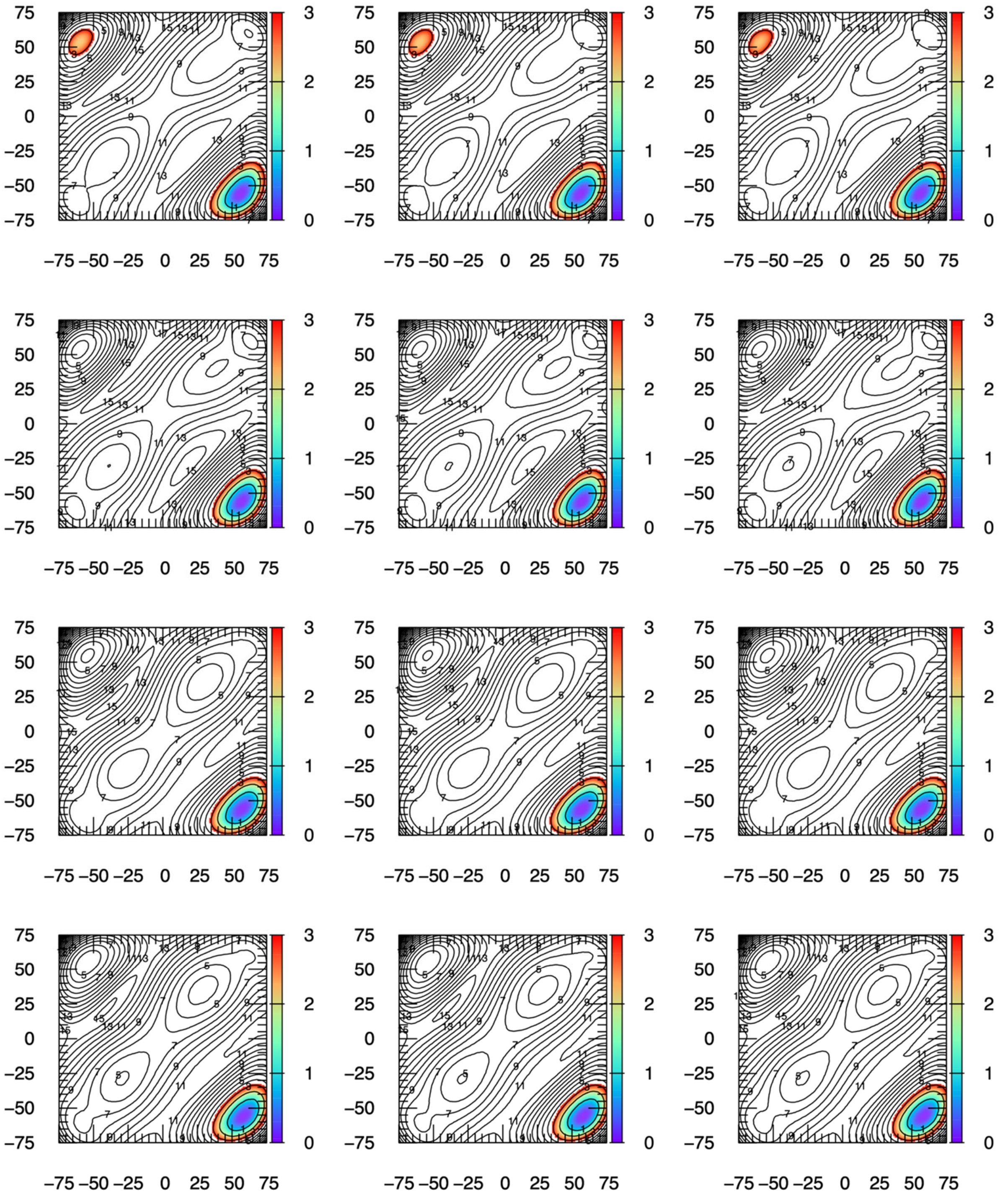

Figure S6. Triplicate eABF $\Delta G(\alpha_1, \alpha_2)$ data for the α- and β-anomers of D-xylose (Xyl) and the corresponding O-methyl glycosides. First row: αXyl; second row: MeαXyl; third row: βXyl; fourth row: MeβXyl. $\Delta G(\alpha_1, \alpha_2)$ is in kcal/mol, with contours drawn every 1 kcal/mol, colored from 0-3 kcal/mol, and labeled every 2 kcal/mol.

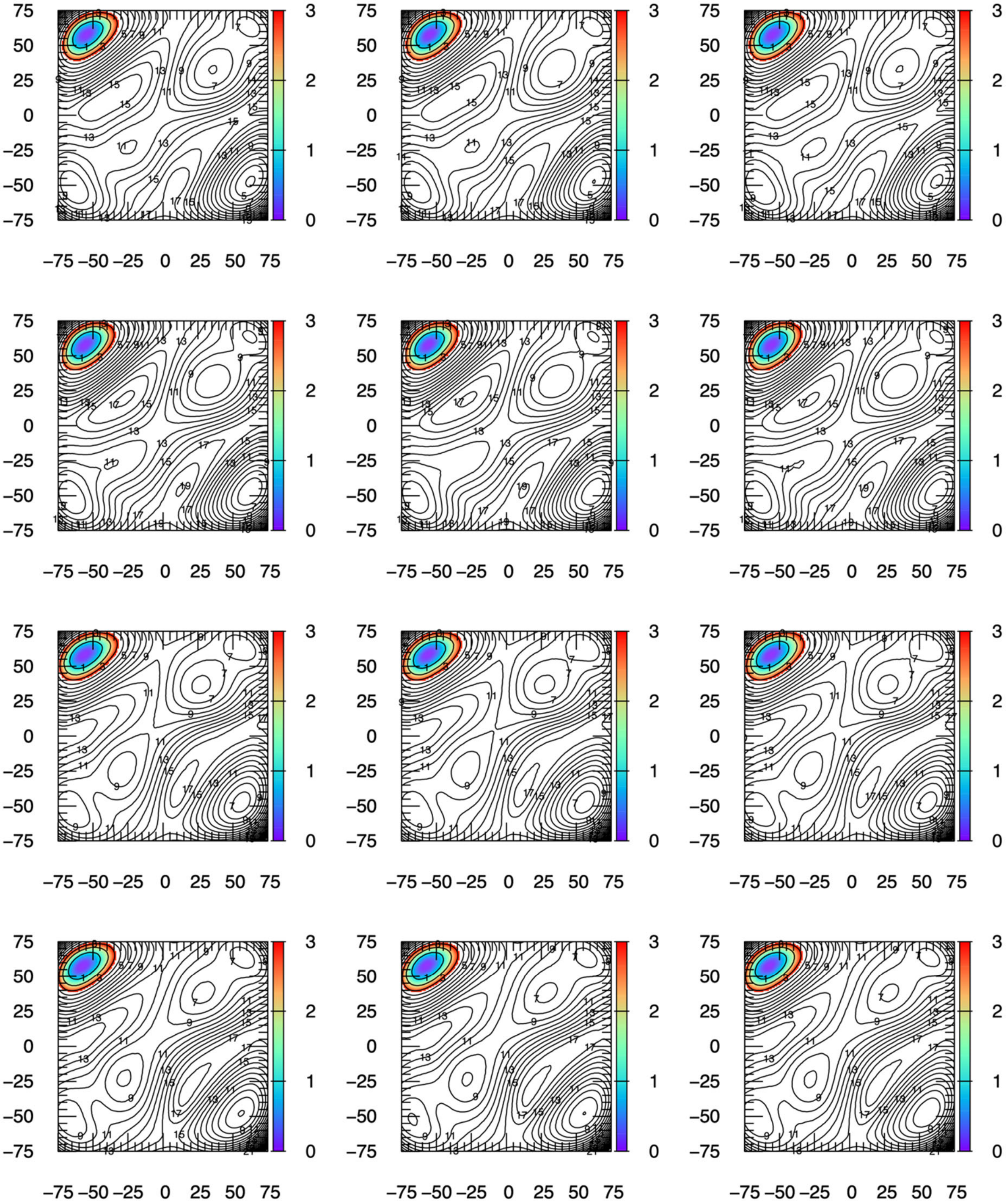

Figure S7. Triplicate eABF $\Delta G(\alpha_1, \alpha_2)$ data for the α- and β-anomers of L-fucose (Fuc) and the corresponding O-methyl glycosides. First row: αFuc; second row: MeαFuc; third row: βFuc; fourth row: MeβFuc. $\Delta G(\alpha_1, \alpha_2)$ is in kcal/mol, with contours drawn every 1 kcal/mol, colored from 0-3 kcal/mol, and labeled every 2 kcal/mol.

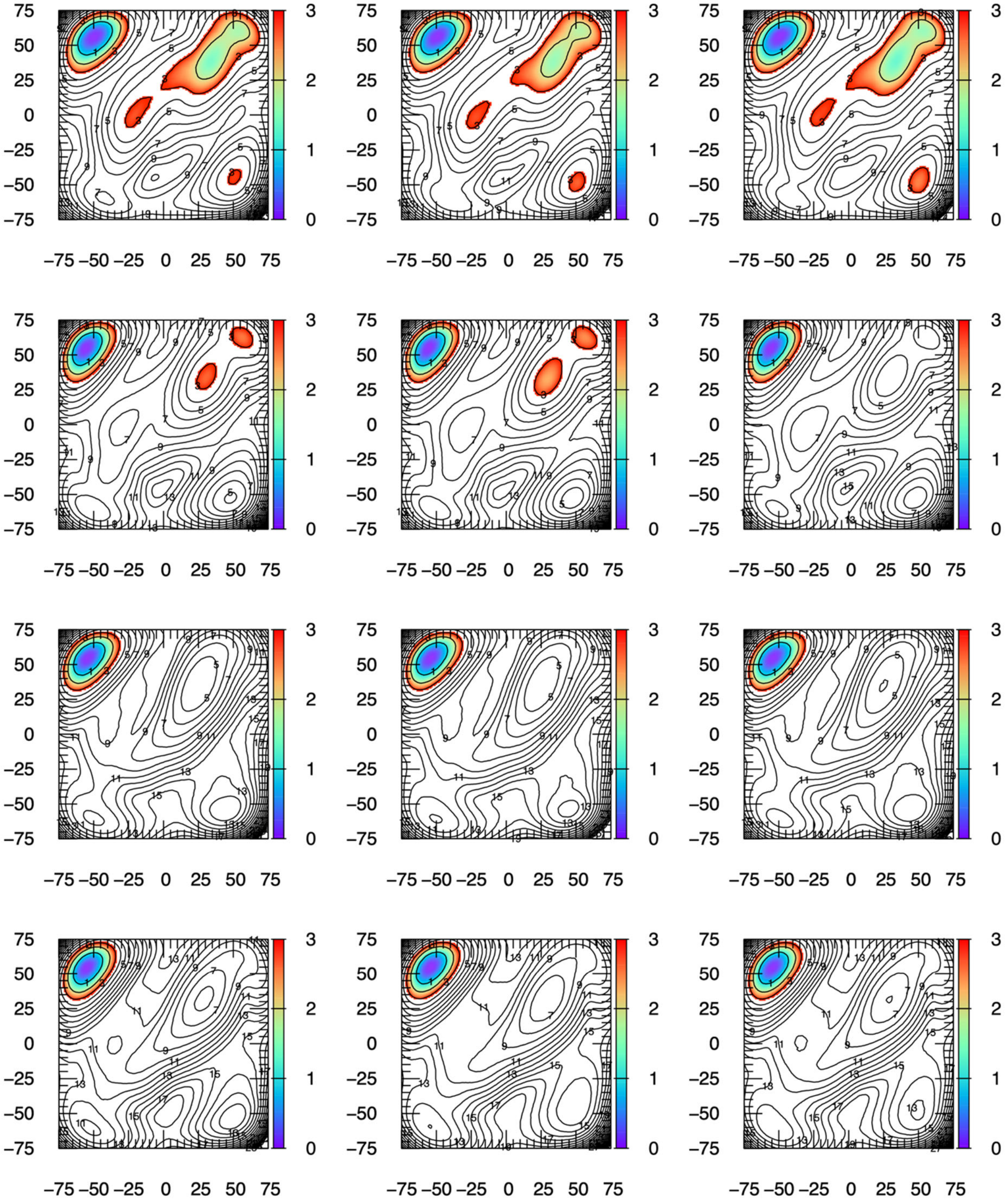

Figure S8. Triplicate eABF $\Delta G(\alpha_1, \alpha_2)$ data for the α- and β-anomers of *N*-acetyl-D-neuraminate (Neu5Ac) and the corresponding O-methyl glycosides. First row: αNeu5Ac; second row: MeαNeu5Ac; third row: βNeu5Ac; fourth row: MeβNeu5Ac. $\Delta G(\alpha_1, \alpha_2)$ is in kcal/mol, with contours drawn every 1 kcal/mol, colored from 0-3 kcal/mol, and labeled every 2 kcal/mol.

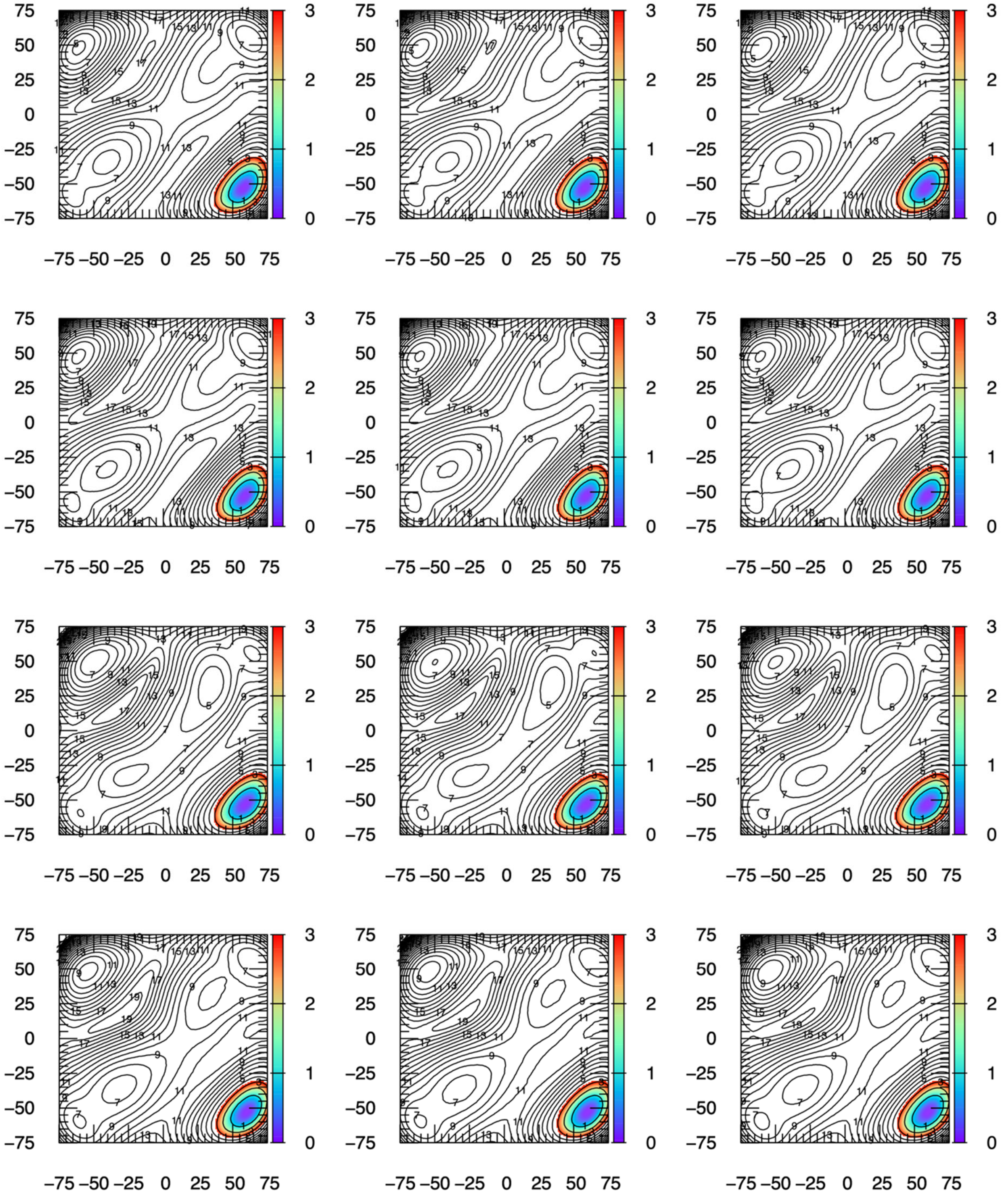

Figure S9. Triplicate eABF $\Delta G(\alpha_1, \alpha_2)$ data for the α- and β-anomers of D-glucuronate (GlcA) and the corresponding O-methyl glycosides. First row: αGlcA; second row: MeαGlcA; third row: βGlcA; fourth row: MeβGlcA. $\Delta G(\alpha_1, \alpha_2)$ is in kcal/mol, with contours drawn every 1 kcal/mol, colored from 0-3 kcal/mol, and labeled every 2 kcal/mol.

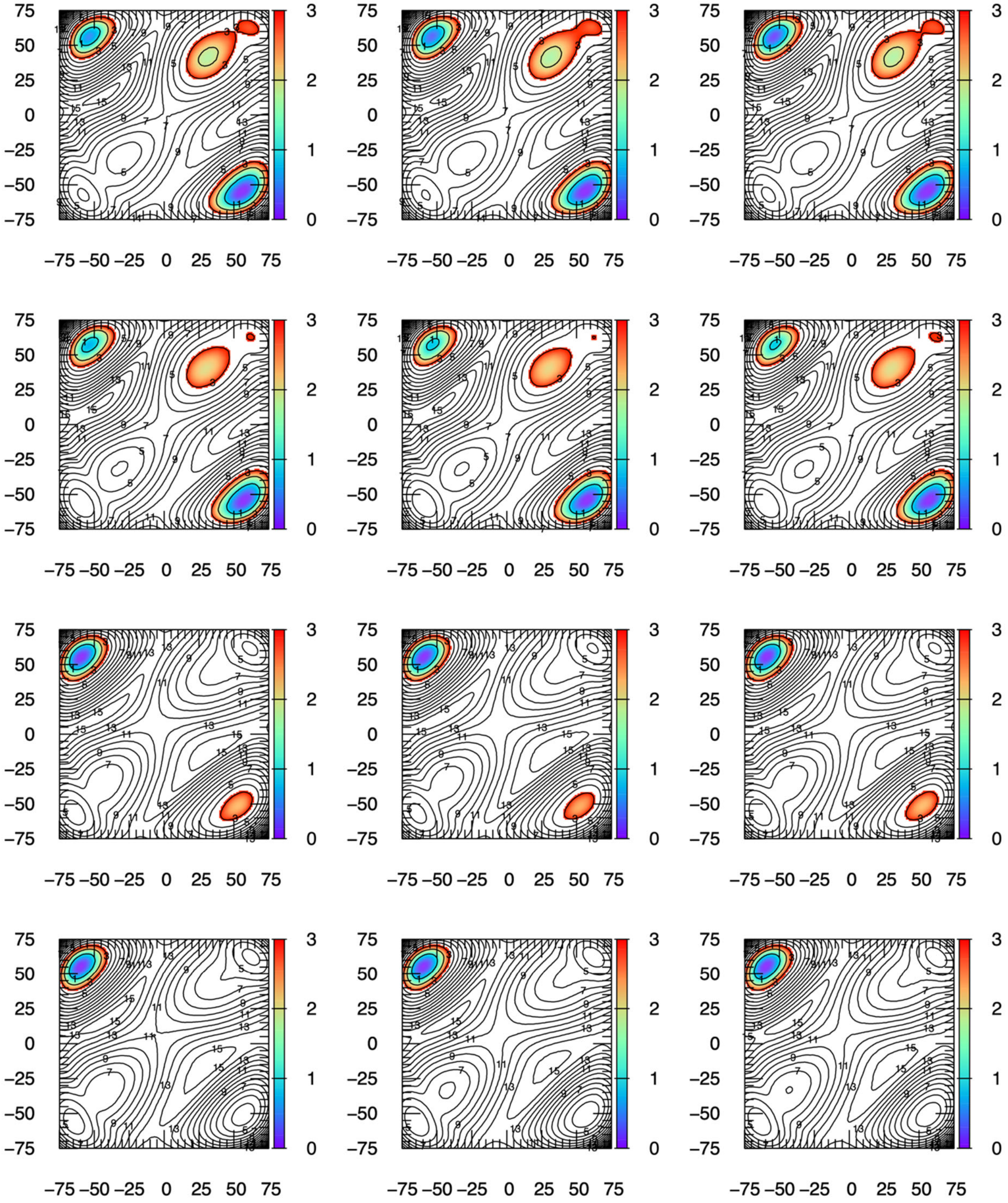

Figure S10. Triplicate eABF $\Delta G(\alpha_1, \alpha_2)$ data for the α- and β-anomers of L-iduronate (IdoA) and the corresponding O-methyl glycosides. First row: αIdoA; second row: MeαIdoA; third row: βIdoA; fourth row: MeβIdoA. $\Delta G(\alpha_1, \alpha_2)$ is in kcal/mol, with contours drawn every 1 kcal/mol, colored from 0-3 kcal/mol, and labeled every 2 kcal/mol.

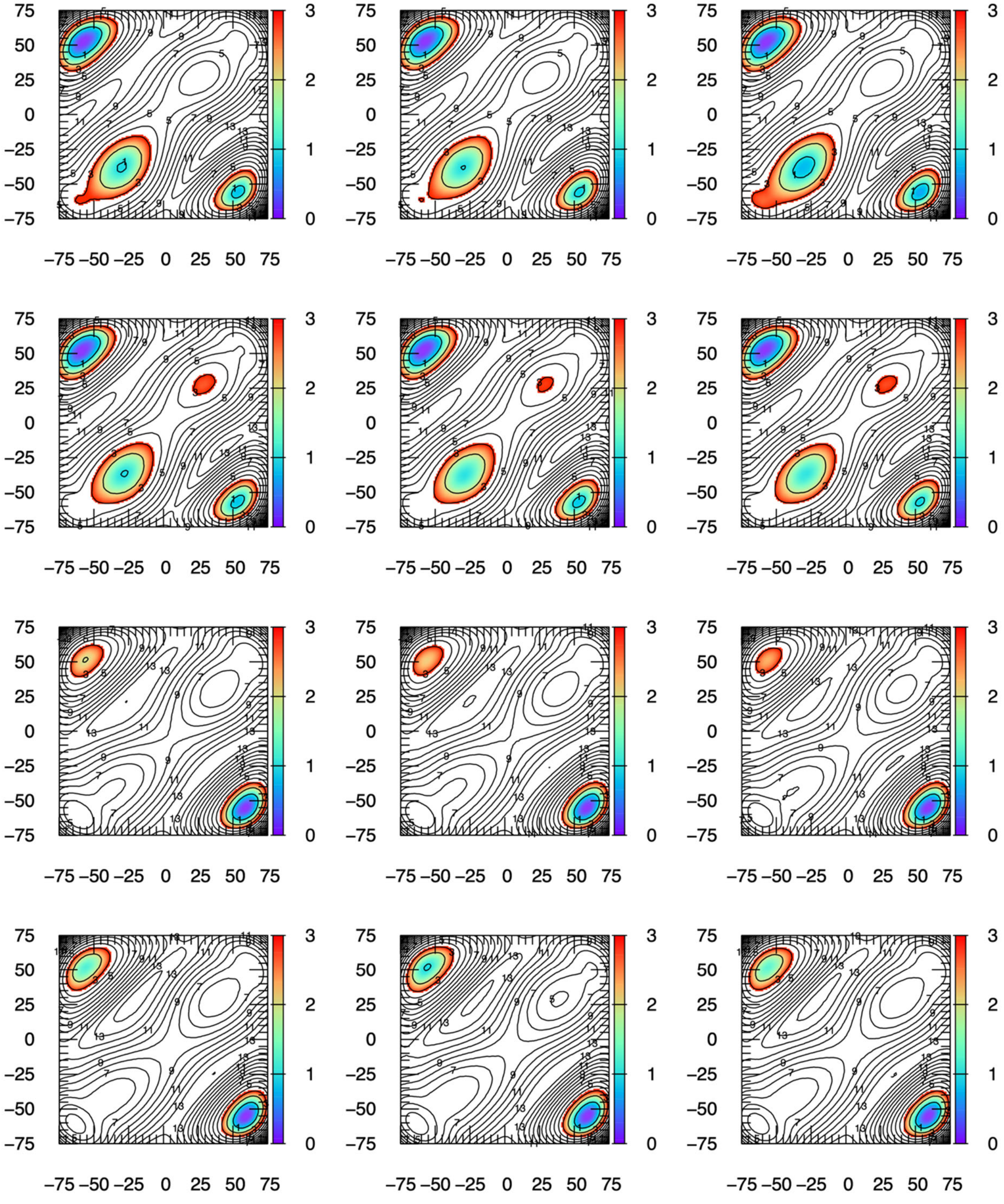

Figure S11. Triplicate eABF $\Delta G(\alpha_1, \alpha_2)$ data for the α- and β-anomers of D-idose (Ido) and the corresponding O-methyl glycosides. First row: αIdo; second row: MeαIdo; third row: βIdo; fourth row: MeβIdo. $\Delta G(\alpha_1, \alpha_2)$ is in kcal/mol, with contours drawn every 1 kcal/mol, colored from 0-3 kcal/mol, and labeled every 2 kcal/mol.

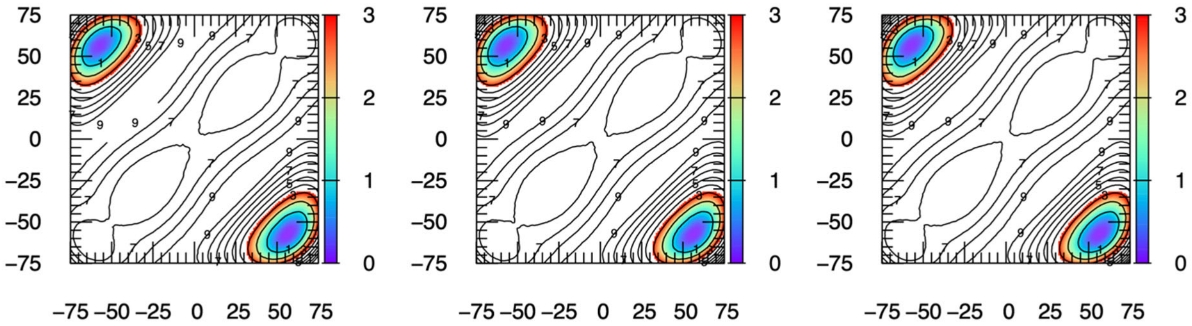

Figure S12. Triplicate eABF $\Delta G(\alpha_1, \alpha_2)$ data for tetrahydropyran (THP). $\Delta G(\alpha_1, \alpha_2)$ is in kcal/mol, with contours drawn every 1 kcal/mol, colored from 0-3 kcal/mol, and labeled every 2 kcal/mol.
